# Query-based biclustering of gene expression data using Probabilistic Relational Models

**DOI:** 10.1186/1471-2105-12-S1-S37

**Published:** 2011-02-15

**Authors:** Hui Zhao, Lore Cloots, Tim Van den Bulcke, Yan Wu, Riet De Smet, Valerie Storms, Pieter Meysman, Kristof Engelen, Kathleen Marchal

**Affiliations:** 1 Microbial and Molecular Systems, K.U.Leuven, Leuven, 3001, Belgium; 2 i-ICT, Universitair Ziekenhuis Antwerpen, Edegem, 2650, Belgium

## Abstract

**Background:**

With the availability of large scale expression compendia it is now possible to view own findings in the light of what is already available and retrieve genes with an expression profile similar to a set of genes of interest (*i.e.*, a query or seed set) for a subset of conditions. To that end, a query-based strategy is needed that maximally exploits the coexpression behaviour of the seed genes to guide the biclustering, but that at the same time is robust against the presence of noisy genes in the seed set as seed genes are often assumed, but not guaranteed to be coexpressed in the queried compendium. Therefore, we developed *Pro*Bic, a query-based biclustering strategy based on Probabilistic Relational Models (PRMs) that exploits the use of prior distributions to extract the information contained within the seed set.

**Results:**

We applied *Pro*Bic on a large scale *Escherichia coli* compendium to extend partially described regulons with potentially novel members. We compared *Pro*Bic's performance with previously published query-based biclustering algorithms, namely ISA and QDB, from the perspective of bicluster expression quality, robustness of the outcome against noisy seed sets and biological relevance.

This comparison learns that *Pro*Bic is able to retrieve biologically relevant, high quality biclusters that retain their seed genes and that it is particularly strong in handling noisy seeds.

**Conclusions:**

*Pro*Bic is a query-based biclustering algorithm developed in a flexible framework, designed to detect biologically relevant, high quality biclusters that retain relevant seed genes even in the presence of noise or when dealing with low quality seed sets.

## Background

With the large body of publicly available gene expression data, compendia are being compiled that assess gene expression in a plethora of conditions and perturbations [1]. Comparing own experimental data with these large scale gene expression compendia allows viewing own findings in a more global cellular context. To this end query-based biclustering techniques [2-6] can be used that combine both gene and condition selection to identify genes that are coexpressed with genes of interest (*i.e.*, a query or seed set, containing one or more genes) for a subset of conditions. These biclustering algorithms do not only differ from each other in their search strategy, but also in the way they exploit the expression signal of the seed genes to identify the query-based biclusters. Some algorithms use the mean expression profile of the seeds to initialize the biclustering [3], while others use the ‘similarity’ between the mean profiles of the seed set and the bicluster to constrain the search at each iteration [5].

For a query-based biclustering algorithm, it is naturally important to keep a bicluster centered around the seed genes as it should not converge to a bicluster that no longer contains the seed genes. However, it can not be guaranteed that all genes within the seed set will be tightly coexpressed. In such cases, adhering too strictly to the query (*e.g.*, by relying heavily on the mean query profile to steer the biclustering), will deteriorate the results as the algorithm will not be able to compensate for incoherent query profiles. An efficient query-based biclustering algorithm should therefore always retain part of the seed genes, but simultaneously allow sufficient freedom to adjust for non-perfect or noisy sets of seed genes. In order to accommodate for these contrasting requirements in a flexible way, we developed a query-based biclustering method called *Pro*Bic. The model is formulated in the framework of Probabilistic Relational Models (PRMs) [7-9]. Query information is exploited via a Bayesian prior. We compared our algorithm with two of the best state-of-the art query-based biclustering algorithms, namely Iterative Signature Algorithm (ISA) [3] and Query-Driven Biclustering (QDB) [5], for a number of different bicluster comparison criteria on a large compendium of *Escherichia coli* microarray experiments.

## Methods

### Model framework

The general goal is the identification of sets of genes with similar expression profiles (coordinated changes) that differ significantly from the background profile in a subset of experimental conditions (*i.e.*, constant column biclusters using the terminology of Madeira and Oliveira [10]). By exploiting information contained in a given set of seed genes, we constrain the search space to biclusters that represent patterns similar to the seed gene pattern.

To this end we developed a framework based on Probabilistic Relational Models (PRMs) for query-driven biclustering of microarray data. PRMs were developed as an extension of Bayesian networks to the relational domain.

**Figure 1:**
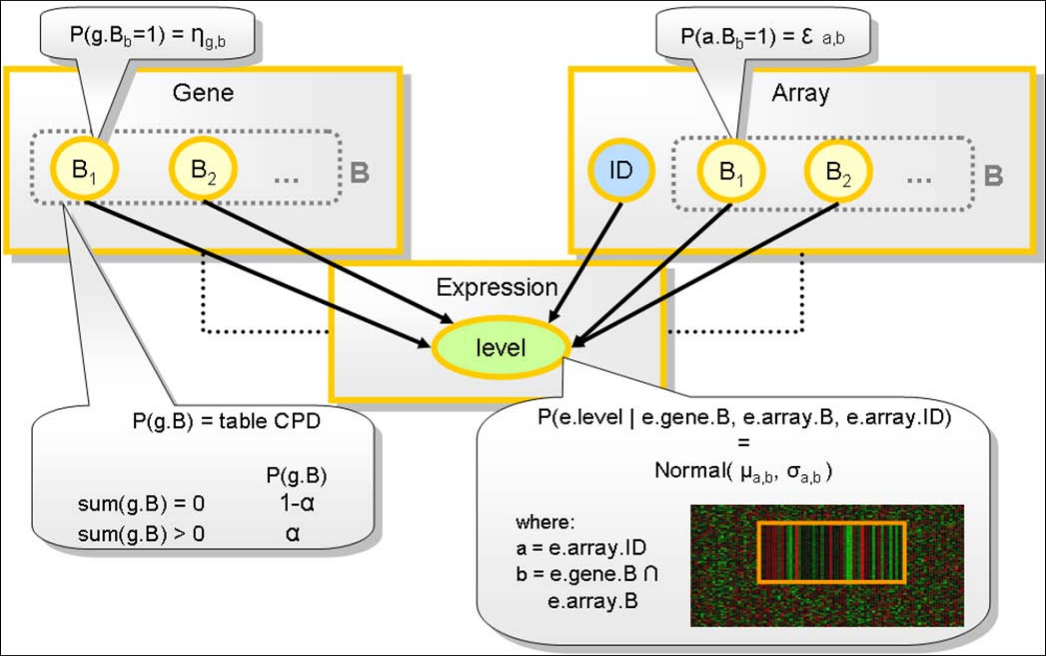
**Schematic overview of the *Pro*Bic model and the conditional probability distributions of the attributes**. Gene, Array and Expression represent the three *Pro*Bic classes of the PRM model. For each class, a set of specific gene, array and expression objects exists (denoted by the lowercase letters *g*, *a* and *e* respectively). The complete set of genes, array and expression objects that belong to a certain class are indicated by uppercase letters *G*, *A* and *E*. For the Gene (Array) class, a Boolean attribute *B_b_* indicates whether a gene (array) belongs to a bicluster *b* or not. For each gene (array) object, the gene-bicluster labels *g.B_b_* (over all biclusters b) and the array-bicluster labels *a.B_b_* are the hidden variables of the model. Each object *e* of the Expression class has one single numeric attribute *e.level* that contains the expression level for each specific gene and array combination. The array class has an additional attribute ID that uniquely identifies each individual array object *a*. The conditional probability distribution *P*(*e.level|e.gene.B*,*e.array.B*,*e.array.ID*) is modeled as a set of Normal distributions, one for each array-bicluster combination. A number of marginal distributions *P*(*a.B_b_*), *P*(*g.B_b_*) and *P*(*g.B*) allow expert knowledge to be introduced in the model, as explained in the main text.

An overview of the *Pro*Bic Probabilistic Relational Model is shown in Figure 1: it contains the classes Gene, Array and Expression. For each class, gene, array and expression objects exist that are specific instantiations of the class (denoted by the lowercase letters *g*, *a* and *e* respectively). The complete set of genes, array and expression objects that belong to a certain class are indicated by uppercase letters *G*, *A* and *E*. Each object *g* and *a* of respectively the Gene and Array class has a number of binary attributes. The Boolean attributes *B_b_* indicate for each gene (array) object whether that gene (array) belongs to a bicluster *b* or not. The gene-bicluster labels *g.B_b_* (over all biclusters *b*) and the array-bicluster labels *a.B_b_* are the hidden variables of the model. The Array class has an additional attribute ID that uniquely identifies each individual array object *a*. This is needed because *Pro*Bic searches for constant column biclusters. Finally, each object *e* of the class Expression has a single numeric attribute *e.level* that contains the expression level for each specific gene and array combination.

The PRM imposes that each expression value that belongs to the same bicluster and array combination is modeled by a distribution with the same parameters.

#### ***Posterior distribution***

The posterior distribution for the *Pro*Bic model is shown in Equation (1).(1)

with θ being the collection of model parameters. In the following sections, we will discuss the likelihood and the prior in detail.

#### Likelihood

The likelihood of the *Pro*Bic model is shown in Equation (2). It expresses how probable the observed dataset is for different settings of the parameter vector θ. For notational convenience, the dependency on the model parameters is not written explicitly.(2)

As shown in Equation (2), this likelihood consists of a conditional probability distribution (CPD) for the expression values and several marginal distributions modeling the assignment of genes and arrays to biclusters (see also following sections).

#### Modeling the expression values

The CPD for modeling the expression values consists of two factors:(3)

The first factor describes the conditional probability of all expression levels given their gene and array bicluster assignment attributes:(4)

A bicluster is modeled by a set of independent Normal distributions, one for each array that was assigned to the bicluster. For each array, an individual expression level is either part of (one or more) bicluster(s) or part of a background distribution. The distribution of the background expression values is modeled per array as a Normal distribution with parameters (*µ_a_*_,_*_bgr_*, *σ_a_*_,_*_bgr_*). The parameters of the background distributions are fixed and derived *a priori* from the dataset using a robust estimation [11].

A second factor regulates the model complexity by adjusting the probability that an expression value is belonging to a bicluster distribution compared to the background distribution.(5)

The parameter that regulates this probability can be defined as a penalty factor log(π_bicl_/ π_bgr_) to control model complexity. The factor indicates how many times more likely it must be that an expression value is part of the bicluster distribution compared to being part of the background distribution before it is actually assigned to that bicluster. Detailed explanations of each of these factors can be found in the Additional File 1: *Detailed explanation of the expression level CPD*.

#### Marginal distributions modeling the assignment of genes and arrays to biclusters

The likelihood function (Equation (2)) contains a number of factors which can be used to introduce expert knowledge.

The probability for the gene to bicluster assignments *P*(*g.B*) is defined as a combination of two factors where each one defines a separate aspect of the prior [11]:(6)

The first factor *P_1_*(*g.B*) reflects general expert knowledge on gene to bicluster assignments. It expresses the prior probability or expectation that a gene will belong to a bicluster, irrespective of the bicluster identity. It expresses our belief in the degree of modularity of the dataset and indirectly affects the average number of genes in a bicluster. By penalizing the addition of genes to a bicluster, one can control the tightness of coexpression in the bicluster. The second factor *P_2_*(*g.B_b_*) reflects prior knowledge on specific gene to bicluster assignments, namely, the probability for a specific gene *g* to belong to a particular bicluster *b*. This prior can be used to introduce detailed biological prior knowledge in the model on genes that should belong together in a bicluster.

Similarly *P*(*a.B_b_*) describes the prior probability for a specific array *a* to belong to a specific bicluster *b*. This prior can be used in a similar way as *P_2_*(*g.B_b_*), namely to introduce prior knowledge, by specifying the conditions that are more likely to belong together.

Each array is also given a unique identifier *a.ID* and in principle a probability distribution can be defined for every array. This distribution was chosen uniform for the analyses described in this study.

#### Prior for the model parameters P(θ)

A bicluster is modeled by a set of independent Normal distributions, one for each array that was assigned to the bicluster. Each bicluster-array combination is thus modeled as a Normal distribution with its own set of parameters (*µ_a_*_,_*_b_*, *σ_a_*_,_*_b_*) (Figure 1). Prior knowledge on the model parameters is introduced in the model through the appropriate prior distributions. We choose for conjugate prior distributions as these result in a simple decomposition of the total probability distribution.(7)

Any member of the exponential family can be used as a conjugate to the distribution of Equation (7). We use Normal-Inverse-χ^2^ priors on the column-wise Gaussian probability distributions. This distribution is parameterized by the hyperparameters (*µ_0_*; *κ_0_*; *ν_0_*; *σ^2^_0_*). *µ_0_* reflects the prior mean and *σ^2^*/ *κ_0_* reflects the *a priori* variance on this mean. The parameters *ν_0_* and *σ^2^_0_* determine the *a priori* variance of the distribution and its associated variance.

The query-based aspect of our biclustering approach exploits the possibility to use strong priors on the bicluster distribution. By choosing the average expression value per array *a* of the set of query genes (µ_a_^query^ ) as the prior mean µ_a,b_^0^ , the algorithm will identify a bicluster *b* that remains centered around the original expression profile of the query genes. The prior standard deviation σ_a,b_^0^ is by default chosen to be smaller than the background standard deviation σ_a_^bgr^ by a fraction *f_bcl_* in order to identify tight bicluster profiles.

To prevent the bicluster from drifting too far away from the seed profile, the parameter κ_0_ should have a high value relative to the other hyperparameters (*i.e.*, to force the variance on the prior mean to be small). The parameter ν_0_ determines the relative weight of the prior versus the data.

A more detailed explanation of the hyperparameters can be found in Additional File 2: *Conjugate prior distributions*.

### Learning the model

To learn the model, we applied a hard-assignment EM approach [12] consisting of the following steps until convergence (for a detailed explanation, see [11]):

• Maximization step: maximize over the model parameters, keeping the hidden variables (*i.e.*, the gene and array bicluster assignments *g.B* and *a.B*) fixed.

• Expectation step: find the expected values for the hidden variables *g.B* and *a.B*, keeping the current model parameters fixed.

As an initialization of the hidden variables (the gene to bicluster and array to bicluster assignments) a set of seed genes is used:(8)(9)

### Datasets

For benchmarking we used a real dataset. This allows for a more unbiased comparison than *e.g.*, the often used Prelic dataset [13] of which the assumptions underlying the data simulation favours some algorithms over others. Real data consisted of an *E. coli* cross-platform expression compendium [14] and sets of seed genes were derived from RegulonDB (version 6.2) [15]. RegulonDB enlists for the documented *E. coli* transcription factors (TFs) both single (all targets regulated by one specific TF) and complex regulons (targets regulated by a specific combination of TFs). We obtained in total 225 different sets of seeds ranging in size from 1 gene to 98 genes, corresponding to 89 simple regulons and 136 complex regulons (see Additional File 3: *Biological dataset*).

### Running *Pro*Bic and benchmarking with other algorithms

For *Pro*Bic, two parameters that are influential in a query-based setting, *i.e.*, *f_bcl_* and log(π_bicl_/ π_bgr_) were tuned. These parameters determine the bicluster size and quality as is illustrated in Additional File 4: *Influence of parameter settings*. ISA was obtained from [16]; QDB was obtained from [17]. For details on the used running parameters for all algorithms, see Additional File 5: *Running parameters of query-based biclustering tools*.

### Calculation of bicluster comparison criteria

In order to assess the quality of the obtained biclusters and compare the results of different algorithms, we define several performance criteria related to both the expression quality of the biclusters as well as the retrieved biclusters' biological relevance.

#### Bicluster expression quality

High quality biclusters are identified as those that contain genes that are mutually tightly coexpressed and that preferentially vary largely over the selected conditions (*i.e.*, with a profile different from the background profile). This behaviour is reflected in two measures: the standard deviation within conditions (STD-within, Equation (10)), and the uncentered standard deviation of the mean profile across conditions (STD-across, Equation (11)) respectively. These two measures can also be summarized in their ratio (*i.e.*, STD-across/STD-within) to objectively assess the expression quality of a bicluster.(10)(11)

In Equation (10) and (11), *G* is the number of genes in the seed set or bicluster, *C* is the number of conditions in the compendium in case of a seed set and the number of conditions selected after biclustering in case of a bicluster. *x_i_*_,_*_j_* is the expression value measured for gene *i* and condition *j*, and  is the mean expression value of the genes in the seed set or bicluster for a certain array *j*.

#### Functional and motif enrichment

For the calculation of the functional enrichment, GO categories were derived from Ecocyc [18]. For the overrepresentation of regulatory motifs in biclusters genome wide motif hits were obtained by motif screening. Screening was performed using Clover [19] and the PWMs representing the motifs of interest. For all first genes of transcription units their upstream region (400bp) was screened. In case of multiple TUs per gene, the TU for which the corresponding first gene had the longest intergenic region was selected. All sequence information was retrieved from NCBI (NC_000913) [20]. We estimated from all motif screening scores a robust noise distribution and used this distribution to select for each motif a threshold on the score. Hits with a score higher than this threshold were considered true motif hits. Seed genes were excluded from the biclusters for all enrichment calculations. Enrichments were calculated using the hypergeometric distribution (0.05 significance level). Due to the discreteness of the distributions one-sided mid-P-values were used [21].

## Results

We used *Pro*Bic to search for genes that are tightly coexpressed with known regulon members in *E. coli*. Genes found to be closely expressed with the regulon seed genes are assumed to be potential undocumented targets for the regulon’s associated TF(s). For all tests described below, we benchmarked our method with other query-based biclustering algorithms for which a high performance on real datasets was shown previously, *i.e.*, QDB [5] and ISA [3]. The results section is divided into two main parts: first we evaluate the performance of the algorithms for retrieving good quality biclusters, next we see whether these perfornance differences actually lead to more biologically relevant results.

### Performance of the algorithms

To evaluate the query-based biclustering performance of the different approaches we assess to what extent the biclusters remain centered around the seed genes, how their expression quality relates to the expression quality of the seed genes, and the ability of the methods to handle noisy seed genes.

#### Behavior towards seed genes

A first illustration of how the used algorithms cope differently with the seed genes is given in Additional File 6: *Behavior of the different algorithms towards seed genes*. This table shows to what extent the final biclusters still contained the original seed genes. It suggests that when a seed set is informative for the queried expression dataset (meaning that the dataset indeed contains additional genes being coexpressed with the seed set), all algorithms are able to find biclusters that contain all or part of the original seed genes. In contrast seed sets that are non-informative for the queried dataset give rise to either ‘empty’ biclusters, to biclusters with only the seed genes or to biclusters that drift away and do not longer contain the seed genes. Here ISA mainly results in biclusters that loose their seeds genes. The reason is that ISA is query-based only in its initialization. Contrary to ISA, *Pro*Bic and QDB both use prior distributions to keep the bicluster profile centered around the seed gene profile.

#### Expression quality of the biclusters

**Figure 2:**
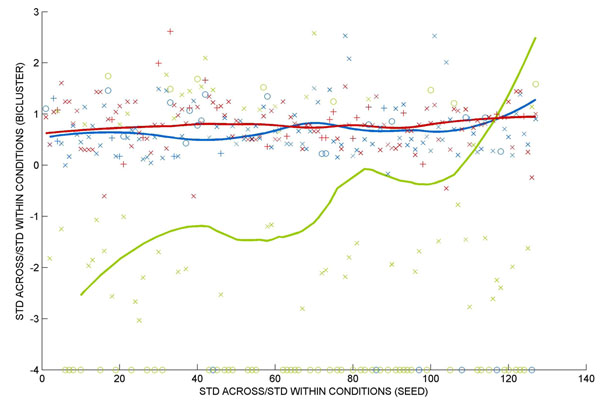
**Effect of the seed set quality on the quality of the retrieved biclusters.** (x-axis) STD-across/STD-within conditions of the seeds, (y-axis) STD-within/STD-across (log2 transformed) of the retrieved biclusters. Bicluster results of respectively *Pro*Bic (blue) – QDB (green) – ISA (red) are subdivided in the following groups (indicated by the respective markers): ‘x’ seed sets that give rise to biclusters that contain the full seed set or part of the seed set together with additional genes, ‘+’ seeds that give rise to biclusters that do not longer contain the seed sets (drift away biclusters), ‘o’ seed sets that give rise to ‘empty’ biclusters or biclusters that contain seed genes only. Only the results of 127 seed sets of which the seed quality could be calculated (seed set should contain more than one seed gene), are displayed. Full lines display the overall trend.

This difference in outcome of *Pro*Bic, QDB and ISA as a function of the seed set properties is further illustrated by plotting the quality of the obtained biclusters as a function of the quality of the seed set on the bicluster results (Figure 2). Both *Pro*Bic and ISA identify biclusters of high expression quality and are much less sensitive towards quality changes in the seed genes than QDB.

#### Difference in handling noisy seed genes

**Figure 3:**
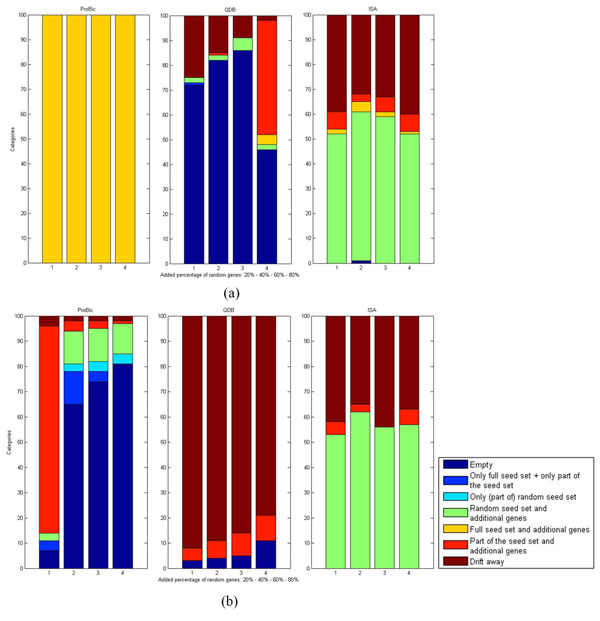
**Effect of noisy seed genes on bicluster results**. Analysis of biclusters, obtained by the different algorithms after biclustering in the presence of an increasing number of random genes (1 = 20%, 2 = 40%, 3 = 60% and 4 = 80% of random genes) added to the true seed set of one seed set of high quality (panel a: ArcR_FadR) and one seed set of low quality (panel b: NagC). The procedure of adding random sets was repeated 100 times for each seed set. Obtained biclusters were categorized according to their behavior with respect to the true seed genes, using the categories ‘Empty’ biclusters, ‘Only full seed set’ biclusters (containing all initial seed genes but without additional genes) and ‘Only part of the seed set’ biclusters (containing only a part of the initial seed genes but without additional genes), ‘Only (part of) random seed set’ biclusters (containing only (a part of) the random genes that were initially added to the true seed set), ‘(Part of) random seed set and additional genes’ biclusters (containing a (part of) the random genes that were initially added to the true seed set and extra genes that were not a part of the initial seed set), ‘Full seed set and additional genes’ biclusters (containing all seed genes together with additional genes), ‘Part of the seed set and additional genes’ biclusters (containing a part of the initial set of seed genes together with additional genes), and ‘Drift away’ biclusters (clusters containing one or more genes, but none of the seed genes).

To systematically analyze the robustness of the different algorithms against the presence of noisy genes in a seed set, we designed experiments for which a certain number of random genes were added to one seed set of ‘high’ quality (*i.e.*, ArcR_FadR), three seed sets of ‘intermediate’ quality (*i.e.*, FadR, NarL and OmpR), and one seed set of ‘low’ quality (*i.e.*, NagC). In the absence of noisy genes, each of these seed sets was shown to result in biologically relevant biclusters representing the regulons from which the seed sets where derived (as assessed by their functional and motif enrichment) for all algorithms. We repeated this procedure of adding random genes hundred times per seed set and assessed to what extent the different algorithms were able to remove these noisy seed genes from the complete seed set in order to retrieve a bicluster that was centered around the true seed genes. Figure 3 shows the results for the high and low quality seed genes.

Results for the seed sets of intermediate quality can be found in the Additional File 7: *Robustness of ProBic*, *QDB and ISA to noisy seed genes of intermediate quality*. *Pro*Bic is most robust against the presence of noisy seed genes: in the high quality seed set it tends to keep all true seed genes and finds extra genes irrespective of the percentage of noisy seed genes that was added to the true seed genes, while QDB and ISA fail to identify biclusters containing the true seed genes.

In the case of a low quality seed set, it is harder to distinguish the true seed genes from the randomly added ones. While all algorithms perform worse under these conditions, *Pro*Bic still retrieves part of the seed genes for up to 20% of noise genes. Both ISA and QDB mostly fail to keep the true seed genes in all conditions.

### Biological relevance

In the previous section we showed how *Pro*Bic outperforms QDB and ISA with respect to a set of objective query-based bicluster criteria. In this section we will evaluate whether *Pro*Bic also leads to more relevant biclusters from a biological perspective. The biological relevance of retrieved biclusters is assessed through functional and motif enrichment calculations and a cross-validation experiment.

#### Functional and motif enrichment

**Figure 4:**
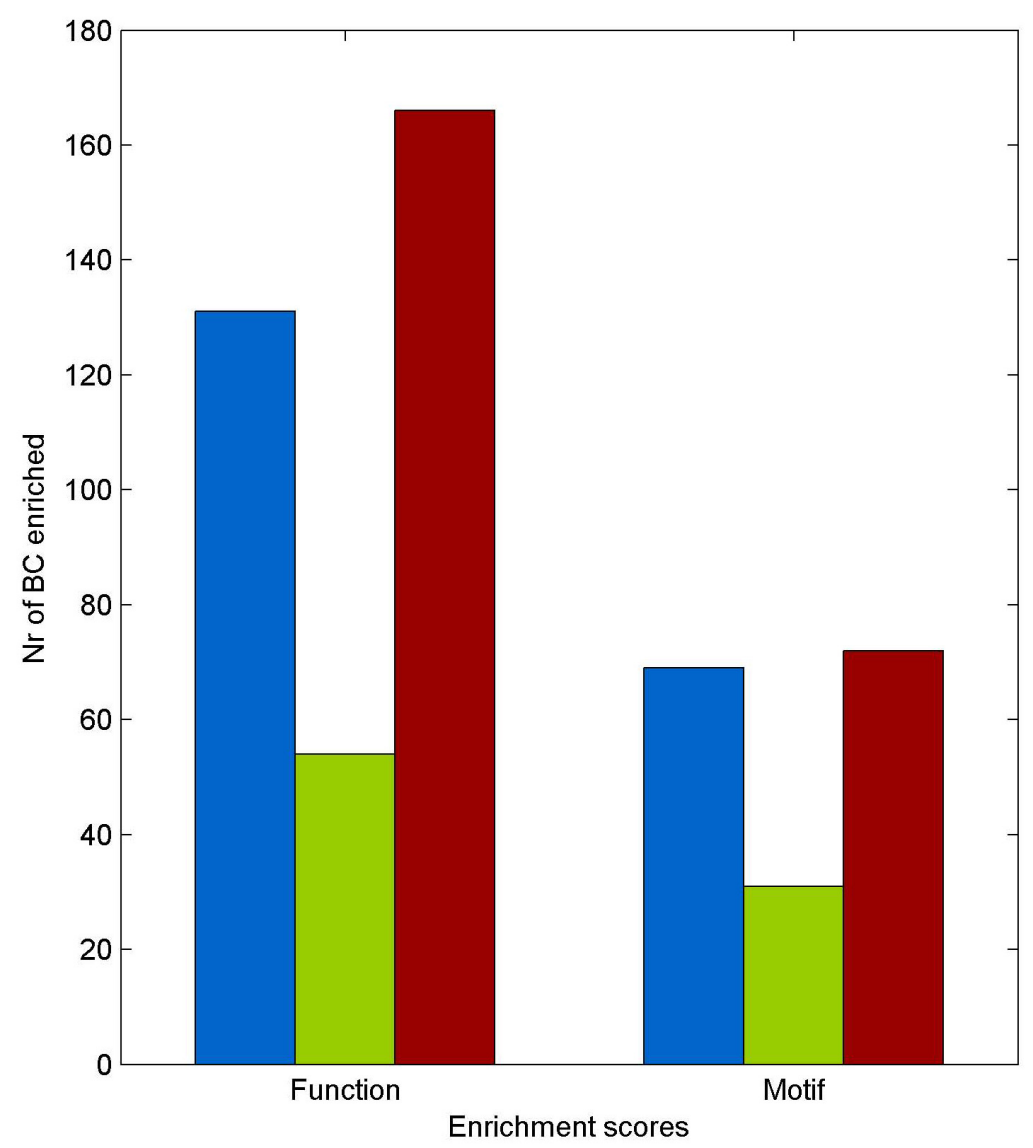
**Biological relevance of the obtained biclusters**. Histogram displaying the percentage of biclusters (derived from a total of 225 different seed sets) that were found to be enriched in ‘functional categories’ that are related to the functions of the original seed genes or TFs, and enriched in ‘motifs’ that represent both the simple and complex regulons from which the seed genes were derived: this indicates to what extent the additionally recruited genes contain similar motifs as the seed genes: *Pro*Bic (blue) - QDB (green) - ISA (red).

To test biological relevance of the biclusters obtained using the 225 seed sets, we assessed to what extent functional classes that were found to be enriched amongst the bicluster genes were similar to the classes to which either the TF of the regulon that was used as seed set or at least one of the seed genes belonged. As an independent assessment of how well the obtained biclusters recapitulate the original simple and complex regulons, we calculated whether our obtained biclusters were overrepresented in the regulatory motifs of the corresponding simple/complex regulons. Figure 4: Biological relevance of the obtained biclusters, shows that both ISA and *Pro*Bic largely outperform QDB at the level of motif and functional overrepresentation. Biclusters retrieved by ISA and *Pro*Bic show a comparable motif enrichment and a slightly better functional enrichment for those derived from ISA than for those obtained by *Pro*Bic: for low informative seeds, *Pro*Bic mainly finds ‘empty’ biclusters or biclusters with only seed genes, whereas ISA drifts away to larger biclusters no longer containing the seeds (see also Additional File 6: *Behavior of the different algorithms towards seed genes*). Both situations gave rise to a similar loss in motif enrichment. Drift away biclusters, while no longer containing the seed genes, can still contain genes that are functionally related to the seed genes in which case they will still contribute to the functional overrepresentation.

#### Cross-validation for identification of known targets of TF(s)

In this experimental set up, we used part of the known regulon members as seeds and tested to what extent the different query-based bicluster approaches could retrieve the left out known targets (*i.e.*, validation set) as additional bicluster members. Of the original 225 regulon sets, we retained the ones with five or more genes. Each of these resulting 49 sets was divided into a seed set and a validation set containing respectively four fifth and one fifth of the number of original seed genes. This procedure was repeated in a five-fold cross-validation set up.

For each of the methods, a query-based biclustering was performed with the seed sets. Results were validated by checking if the left out genes of the original regulon set (*i.e.*, the validation set) were retrieved in the obtained biclusters. To this end we calculated the percentage of genes of the validation set that were retrieved in the biclusters (*i.e.*, recall). To compensate for the fact that the recall is likely to increase with the size of the biclusters, we also calculated the percentage of validation genes found in the bicluster to the total number of genes in the bicluster (*i.e.*, ‘enrichment’ of validation genes in the bicluster). Results presented in Additional File 8: *Recall and ‘enrichment’ of the biclusters in a cross-validation experiment*, are the averages of the five cross-validations and show that for most seed sets, biclusters of *Pro*Bic show a higher recall and enrichment of the genes in the validation set than biclusters obtained by QDB and ISA. In most cases none of the algorithms is able to retrieve all of the validation genes (*i.e.*, a recall of 1). This is to be expected as a regulon membership not necessarily implies coexpression under the conditions of the expression compendium.

## Discussion

In this work we developed *Pro*Bic, a query-based biclustering model formulated in the framework of Probabilistic Relational Models. We compared *Pro*Bic to related query-based biclustering techniques with respect to obtaining high quality and biologically relevant biclusters, centered around the seed genes and with respect to their ability of dealing with noisy seed genes. Although query-based biclustering by ISA results in biologically relevant biclusters of high expression quality, ISA does not constrain the bicluster to remain centered around the seed genes and therefore cannot handle noisy genes in the seed set. *Pro*Bic on the other hand does so in a soft way by using prior distributions to constrain the bicluster distributions. In that sense *Pro*Bic is more similar to QDB, another model based biclustering algorithm. Despite their model similarities, QDB and *Pro*Bic differ in the implementation. Firstly, QDB estimates the parameters for the background distribution for each array during the iterations of the optimization procedure from the expression values on an array that are not yet assigned to a bicluster. However, as genes on the array can be over- or underexpressed without belonging to the current bicluster, the background variance will tend to get overestimated, rendering it more difficult to determine whether for a certain selection of genes a condition belongs to the background or not. *Pro*Bic on the other hand estimates the parameters of the background distributions upfront *and* in a robust way, *i.e.*, by 'filtering out' per condition the most over- and underexpressed genes.

Another major difference is the way the model is learned: whereas *Pro*Bic is run with one parameter setting until convergence to a local optimum, QDB uses a resolution sweep: *i.e.*, the prior variance on the bicluster distributions is increased during several consecutive runs of the algorithm, allowing biclusters to become gradually more coarse grained. The starting point for a new run with an increased prior variance is the solution (*i.e.*, a mode in the posterior landscape) that was found in a previous run performed with a slightly smaller prior variance. Applying strong priors on the model parameters give rise to rather simple posterior distributions and a fast convergence for each prior variance. This can come at the cost of the bicluster quality: relying too heavily on the seed genes explains the bad performance in case of noisy seed genes and the dependence of the quality of the bicluster on the quality of the seed genes.

Because of the ability to use a less informative prior and the characteristics of the estimation of the background distributions, *Pro*bic tends to find biclusters with tightly coexpressed genes and conditions in which the bicluster genes are markedly differentially expressed. This is reflected in the high scores for the quality parameters. Biclusters retrieved by QDB on the other hand are often coarse grained, noisy and large in both genes and conditions.

## Conclusions

*Pro*Bic is a query-based biclustering algorithm, designed to detect biologically relevant, high quality biclusters that retain their seed genes even in the presence of noise or when dealing with low quality seeds. It outperforms QDB and ISA with respect to a set of objective query-based bicluster criteria, namely to what extent the biclusters remain focused around the seed genes, how their expression quality relates to the expression quality of the seed genes, and their ability in handling noisy seed genes. This increased performance also resulted in more relevant biclusters from a biological perspective. In addition, the underlying PRM-based framework is extendable towards integrating additional data sources such as motif information and ChIP-chip information that can further help refining the obtained biclusters.

## Authors' contributions

TVDB and HZ developed the *Pro*Bic model and the query-based extension. PM and KE were responsible for the data acquisition. LC, YW, HZ performed the experiments. VS performed the motif screening. LC, KE, HZ, KM and RDS analyzed the results. LC, KM, HZ, KE and TVDB wrote the manuscript. KM supervised the study. All authors read and approved the final manuscript.

## Competing interests

The authors declare that they have no competing interests.

## Supplementary Material

Additional File 1 **Detailed explanation of the expression level CPD** It contains a detailed explanation of the expression level CPD formulated in the section ‘Model framework’ in the main text. Click here for file

Additional File 2 **Conjugate prior distributions** It contains a detailed explanation of the prior distributions on the model parameters, described in the section ‘Model framework’ in the main text. Click here for file

Additional File 3 **Biological dataset** It contains a table that gives the full list of the seed sets derived from simple and complex regulons and the regulons’ associated TF(s) used in this article to benchmark the different query-based biclustering approaches. Click here for file

Additional File 4 **Influence of parameter settings** It contains an analysis of the influence of two parameters that are influencial in a query-based setting on the obtained bicluster result for five representative seed sets. Click here for file

Additional File 5 **Running parameters of query-based biclustering tools** It contains the parameter settings for all query-based biclustering algorithms (*Pro*Bic, QDB and ISA) that were used to run the experiments performed in the article. Click here for file

Additional File 6 **Behavior of the different algorithms towards seed genes** It contains an additional table to the section ‘Behavior towards seed genes’, that displays the number of bicluster results for respectively *Pro*Bic, QDB, ISA belonging to different categories related to the seed genes. Click here for file

Additional File 7 **Robustness of *Pro*Bic, QDB and ISA to noisy seed genes of intermediate quality** It contains additional figures to the section ‘Difference in handling noisy seed genes’, that depict the performance of respectively *Pro*Bic, QDB and ISA in case of noisy genes added to three seed sets of intermediate quality. Click here for file

Additional File 8 **Recall and ‘enrichment’ of the biclusters in a cross-validation experiment** It contains an additional figure to the section ‘Cross-validation for identification of known targets of TF(s)’, that depicts the recall and enrichment scores for the different seed sets, for each of the three query-based biclustering approaches. Click here for file
