# Supplementary material for: Query-based biclustering of gene expression data using Probabilistic Relational Models
Source: BMC Bioinformatics. 2011 Feb 15;12(Suppl 1):S37. doi: 10.1186/1471-2105-12-S1-S37 (PMC3044293; doi:10.1186/1471-2105-12-S1-S37)
Supplement: Additional File 1 — Detailed explanation of the expression level CPD It contains a detailed explanation of the expression level CPD formulated in the section ‘Model framework’ in the main text. [file 1471-2105-12-S1-S37-S1.pdf]

Additional File 1 - Detailed explanation of the expression level CPD

As mentioned in the main text, the main CPD for the biclustering model consists of two individual factors:

$$
\begin{aligned}
&P(e.level \mid e.gene.B, e.array.B, e.array.ID) = \\
&P_1(e.level \mid e.gene.B, e.array.B, e.array.ID) \cdot \\
&P_2(e.level \mid e.gene.B, e.array.B, e.array.ID)
\end{aligned}
\qquad (1.1)
$$

1.1. CPD factor 1: P_1(e.level | e.gene.B, e.array.B, e.array.ID)

This factor describes the main conditional probability that an expression level belongs to a distribution, determined by the gene to bicluster assignment *e.gene.B*, the array to bicluster assignment *e.array.B* and a specific array ID *e.array.ID*. Below we first define the CPD $P_1(\ldots)$ for three specific situations: one in which the expression value was assigned to the background, one in which the expression value was assigned to a single bicluster and one in which the expression value is assigned to different overlapping biclusters. Based on these specific definitions (indicated by a *) we introduce the generalized definition of $P_1(\ldots)$ that covers all three situations.

Situation 1: background distributions

If the expression level is not part of any bicluster (*e.gene.B* ∩ *e.array.B* = ø), it is assigned to a virtual bicluster with index -1 that describes the background. This bicluster is described with separate Normal distributions ($\mu_a^{bgr}, \sigma_a^{bgr}$), one for each array *a*. The parameters of these distributions are fixed and derived *a priori* from the dataset using a robust estimation.

Situation 2: biclusters without overlap

If no overlap occurs between different biclusters, each expression level can only be assigned to exactly one bicluster, each of which is modeled with Normal distributions with parameters (μ,σ). The values of these parameters depend on the gene to bicluster and array to bicluster assignments (g.*B, a.B*) and on the unique array identifier a.*ID*.

The probability $P_1(\ldots)$ to observe an expression level that belongs to a single bicluster only, is defined as:

$$\begin{aligned} &P_1(e.level \mid e.gene.B, e.array.B, e.array.ID) \\ &= P_1^*(e.level \mid e.array = a, e.bicluster = \{b\}) \\ &= P_1^*(e.level \mid \mu_{a,b}, \sigma_{a,b}) \\ &= \frac{1}{\sigma_{a,b}\sqrt{2\pi}} \exp\left[-\frac{(e.level - \mu_{a,b})^2}{2\sigma_{a,b}^2}\right] \end{aligned} \tag{1.2}$$

We introduced the probability $P_1^*(e.level|e.array = a, e.bicluster = \{b\})$ as the probability that an expression level belongs to a single bicluster. The attribute *e.bicluster* does not formally exist in the model, but it is implicitly defined as the set of bicluster indices to which the expression level belongs, namely the intersection $e.array.B \cap e.gene.B$.

Situation 3: overlapping biclusters

When different biclusters overlap, an expression level can belong to multiple biclusters. To avoid overfitting it seems appropriate to model the overlap region using the parameter sets that were already defined for the individual biclusters (situation 2, *i.e.*, one parameter set per array-bicluster combination). For example, by relying on a definition of the overlap, $P_1(\ldots)$ would be assigned a high probability if the expression levels either approximate the sum, average,

weighted sum, minimum, or the maximum, etc. of the probability distributions in the contributing biclusters.

In our model we choose for an overlap model where the probability of an expression level in the overlap region is defined as the geometric mean of the probabilities assigned to the expression levels based on the distribution of the individual biclusters. For computational reasons, we assumed that the standard deviations of the distributions of the overlapping biclusters are almost identical and that an expression level can maximally belong to two biclusters and. Formally, $P_1(\ldots)$ can then be defined as:

$$\begin{aligned} &P_1(e.level \mid e.gene.B, e.array.B, e.array.ID) \\ &= \prod_{b \in \{iset(e.gene.B) \cap iset(e.array.B)\}} P_1^*(e.level \mid a,b)^{1/\#\{iset(e.gene.B) \cap iset(e.array.B)\}} \\ &= \prod_{b \in iset(B_e^i)} P_1^*(e.level \mid \mu_{a,b}, \sigma_{a,b})^{1/\#iset(B_e^i)} \end{aligned} \quad (1.3)$$

where the following notation is used: *iset(X),* denoting the set of indices *i* for which the vector elements X_i of binary vector *X* are 1. B^i_e is defined as the dot product of *e.gene.B* and *e.array.B*. Therefore, $iset(B^i_e)$ is the set of bicluster-indices in the intersection of *e.gene.B* and *e.array.B*, or formally: $iset(B^i_e) = iset(e.gene.B) \cap iset(e.array.B)$. Finally, $\#iset(B^i_e)$ is the number of elements in this set.

Generalized formula

The following notation covers all situations mentioned above:

$$\begin{aligned} &P_1(e.level \mid e.gene.B, e.array.B, e.array.ID) \\ &= \prod_{b \in iset(B_e^i)} P_1^*(e.level \mid \mu_{a,b}, \sigma_{a,b})^{1/\#iset(B_e^i)} \end{aligned} \quad (1.4)$$

Situation 2 is implicitly covered in the notation of situation 3 as it can be formulated as a special case of 'overlap' with only one bicluster. Situation 1 is covered by the use of the virtual bicluster with index -1. This background bicluster can by definition not overlap with any other bicluster. The definition of the set *iset(*B^i_e*)* is also slightly different from how it was defined in situation 2 as $\prod_{b \in iset(B_e^i)}$ now covers:

- B^i_e empty: background distribution, the product is over the set $b \in [-1]$ so *iset(*B^i_e*)* $= [-1]$.
- B^i_e not empty: bicluster distribution, the product is over the set of biclusters in the intersection and never includes $b = -1$ by definition.

1.2. CPD factor 2: P_2(e.level | e.gene.B, e.array.B, e.array.ID)

Without penalizing for model complexity, the MAP solution would include a very large number of biclusters since each additional bicluster introduces additional degrees of freedom to model the expression values. Models with many biclusters can better explain the data and thus result in higher MAP solutions. Reducing model complexity in a traditional way by including additional terms in the log-likelihood or log-posterior distributions (such as the Bayesian information criterion (BIC) [1] or the Akaike information criterion (AIC) [2]) would lead to computational intractability if an Expectation-Maximization algorithm is used to find the MAP solution. The optimization algorithm assumes independent optimizations per gene or per array in the substeps of the EM procedure. This independency does no longer exist if one of the criteria mentioned above is included in the model.

Therefore, an alternative strategy is used to reduce model complexity by introducing a 'penalty' factor $P_2(\ldots)$. The additional penalty factor $P_2(\ldots)$ is defined such that it only allows a set of

expression levels to be included in a bicluster if they are on average N times more likely to be in their respective bicluster distributions than in their background distributions. The factor $P_2(\ldots)$ decomposes similarly to $P_1(\ldots)$, leading to the following expression:

$$P_2(e.level \mid e.gene.B, e.array.B, e.array.ID) = \prod_{b \in iset_B(e)} P_2^*(e.level \mid b)^{\frac{1}{\#iset_B(e)}} \qquad (1.5)$$

where $P_2(e.level \mid b) = \pi_{bgr}$ describes that probability that the expression level belongs to the background bicluster ($b = -1$) and $P_2(e.level \mid b) = \pi_{bicl}$ describes the probability that the expression level belongs to a bicluster other than the background ($b \neq -1$). This implies that a subset of expression levels E_s for a particular gene or array will be assigned to a bicluster if Equation (1.6) holds:

$$\prod_{e \in E_S} P^*(e, bicl) \cdot \prod_{e \in E_s} \pi_{bicl} > \prod_{e \in E_s} P^*(e, bgr) \cdot \prod_{e \in E_s} \pi_{bgr}$$
$$\Leftrightarrow \qquad (1.6)$$
$$\prod_{e \in E_s} \frac{\pi_{bicl}}{\pi_{bgr}} > \prod_{e \in E_s} \frac{P^*(e, bgr)}{P^*(e, bicl)}$$

The user-defined ratio $\frac{\pi_{bicl}}{\pi_{bgr}}$ indicates how many times more likely it must be on average that an expression value is part of the bicluster distribution compared to being part of the background distribution before such a set of expression values E_s is actually added to the bicluster. To guide the user in determining this ratio, we assume there exists one or more sets of genes in the dataset that are known to be coexpressed. In most practical biological situations, such known sets of genes exist (*e.g.*, a set of operon genes). If such a set would not be available, standard clustering techniques can also be used to identify one or more these clusters. Figure 1.1 illustrates how to choose the ratio $\frac{\pi_{bicl}}{\pi_{bgr}}$ given that a set of genes is known to be coexpressed. We calculate for

every array *a*, the probability that is generated by a bicluster distribution to which it is assigned versus its score of being generated by the background distribution. The difference between these two probability scores, is defined as δ. If the conditions under which these genes are coexpressed also known in advance (see Figure 1.1 (top panel)), we use the known labels that indicate whether or not the arrays belong to the bicluster to train a classifier. This implies determining the optimal threshold of δ so that the global error rate of misclassifying an array with known label is minimized (= the product of the false positive rate and the false negative rate). If the conditions are unknown in advance, a plot of sorted δ's is made. The suggested δ is the one that makes the best distinction between arrays with a low δ and arrays with a high δ value (cut-off point) as shown in Figure 1.1 (bottom panel).

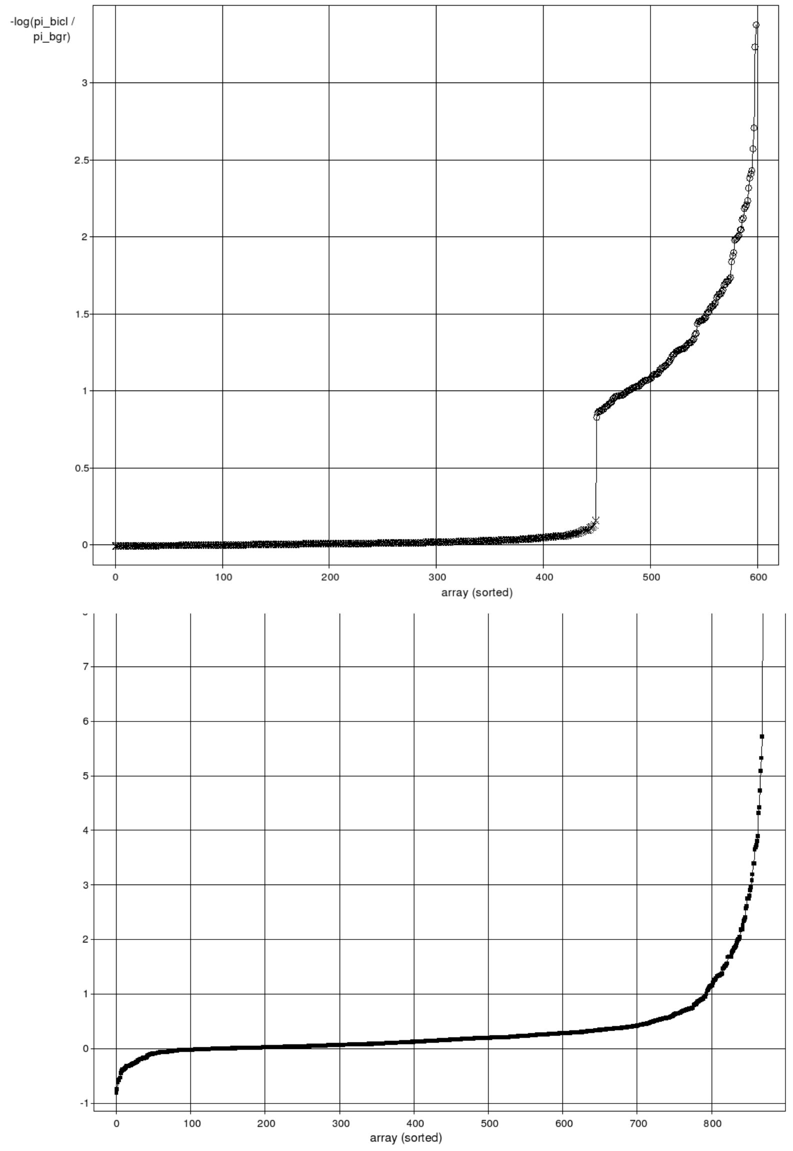

Figure 1.1. Determining the ratio log $\frac{\pi bicl}{\pi bgr}$. (top) Results of a simulated 500x200 dataset with three 50x50 biclusters (noise level 0.2). The plot shows the δ's over all arrays (multiplied with the number of biclusters) for a set of genes that are known to be coexpressed in a number of arrays. Large δ's indicate that the expression levels of the genes are more likely to be part of the bicluster distribution for that array than to be part of the background distribution. The δ threshold that best classifies these two sets of arrays according to the ratio is 0.5, leading to an optimal

ratio of $\log\frac{\pi_{bicl}}{\pi_{bgr}} = -0.5$. (bottom) In the *E. coli* compendium for the set of genes that are known to be regulated by FNR, the plot shows sorted δ's over all arrays. Based on this plot, well chosen values for $\log\frac{\pi_{bicl}}{\pi_{bgr}}$ range between -0.5 and -1.0.

References

1. Schwarz G: **Estimating the Dimension of a Model**. *Annals of Statistics* 1978, **6**:461-464.

2. Akaike H: **A new look at the statistical model indentification**. *IEEE Transactions on Automatic Control* 1974, **19**:716-722.
