# Supplementary material for: Query-based biclustering of gene expression data using Probabilistic Relational Models
Source: BMC Bioinformatics. 2011 Feb 15;12(Suppl 1):S37. doi: 10.1186/1471-2105-12-S1-S37 (PMC3044293; doi:10.1186/1471-2105-12-S1-S37)
Supplement: Additional File 2 — Conjugate prior distributions It contains a detailed explanation of the prior distributions on the model parameters, described in the section ‘Model framework’ in the main text. [file 1471-2105-12-S1-S37-S2.pdf]

Additional File 2 - Conjugate prior distributions

We use the Normal-Inverse-χ^2 distribution as a prior on the parameters of model distribution of the bicluster-array combination ($\mu_{a,b}$,$\sigma_{a,b}$). This defines the following prior distributions for the mean and standard deviation:

$$\sigma \sim Inv-\chi^2(\nu_0,\sigma_0^2) \quad (2.1)$$

$$\mu \mid \sigma \sim N(\mu_0, \sigma^2/\kappa_0) \quad (2.2)$$

This leads to the following formal distribution of the prior which is parameterized by

$(\mu_0,\kappa_{0,}\nu_0,\sigma_0^2)$:

$$P(\mu_{a,b},\sigma_{a,b}) = \sigma^{-1}(\sigma^2)^{-\nu_0/2+1}\exp(-\frac{1}{2\sigma^2}[\nu_0\sigma_0^2+\kappa_0(\mu_0-\mu)^2]) \quad (2.3)$$

The posterior distribution *P(E.level,G.B,A.B,A.ID). P($\mu_{a,b}$,$\sigma_{a,b}$)* is then again a Normal-Inverse-χ^2 distribution, characterized by:

$$\mu_p = \frac{\kappa_0}{\kappa_0+n_X}\mu_0 + \frac{\kappa_0}{\kappa_0+n_X}\frac{s_X}{n_X} \quad (2.4)$$

$$\kappa_p = \kappa_0 + n_X \quad (2.5)$$

$$\nu_p = \nu_0 + n_X \quad (2.6)$$

$$\nu_p\sigma_p^2 = \nu_0\sigma_0^2 + (n_X - 1)ssq + \frac{\kappa_0 n_X}{\kappa_0+n_X}(\frac{s_X}{n_X} - \mu_0)^2 \quad (2.7)$$

As shown in Equation (2.3), four hyperparameters (μ_0,κ_0,ν_0,σ^2_0) determine the prior distribution.
