# Supplementary material for: Query-based biclustering of gene expression data using Probabilistic Relational Models
Source: BMC Bioinformatics. 2011 Feb 15;12(Suppl 1):S37. doi: 10.1186/1471-2105-12-S1-S37 (PMC3044293; doi:10.1186/1471-2105-12-S1-S37)
Supplement: Additional File 3 — Biological dataset It contains a table that gives the full list of the seed sets derived from simple and complex regulons and the regulons’ associated TF(s) used in this article to benchmark the different query-based biclustering approaches. [file 1471-2105-12-S1-S37-S3.pdf]

Additional File 3 - Biological dataset

For the application of *Pro*Bic on biological datasets, a cross-platform compendium for *E. coli* [1] was used, containing 870 publicly available microarrays for diverse experimental conditions. Seed genes were derived from RegulonDB (version 6.2) [2]. RegulonDB contains curated information on experimentally verified regulon membership (a regulon of a TF is defined as the collection of all targets regulated by this TF). RegulonDB makes a distinction between simple and complex regulons. A simple regulon of a TF corresponds to all targets known to be regulated by this TF. A complex regulon corresponds to all targets regulated by the specific combination of TFs. We obtained in total 225 different seed sets ranging in size from 1 gene to 98 genes, corresponding to 89 simple regulons and 136 complex regulons. Table 3.1 gives the full list of the seed sets used in this article.

Table 3.1: List of seed sets derived from simple and complex regulons

TF(s): TFs known to regulate the regulon members are presented in the right hand column. Regulon members: the genes indicated by their b-numbers belonging to regulons regulated by the TFs are presented in the left hand column. Each set of regulon members was used as a separate seed set.

**TF(s)**	**Regulon members**
ArcA_PdhR	b0432
GalR_GalS	b2943,b0759
Ada	b2068,b2213,b4187
CRP_Fis	b4000,b3588,b0440,b4111,b3357,b4069,b3723,b3171,b1101
CRP_GlpR	b2241,b3426,b2240
DcuR_FNR_NarL	b4154
GcvA	b4443,b2808,b2905
TrpR	b1265,b4393,b1704,b3161,b0388
AtoC_IHF	b2221
LexA	b2699,b1728,b1183,b0958,b0799,b3065,b2616,b4058,b4059
SgrR	b2379,b0069,b4577

CRP_FNR	b2957,b0034,b0904,b1182
GlcC	b2980,b2979
HcaR	b2538,b2537
MarA_Rob_SoxR_SoxS	b1611
CRP_CpxR_CytR	b3363
CysB	b1987,b2752,b2425,b2414,b1379,b0365,b0937
FNR_PdhR	b0113,b1109
AlaS	b2697
CRP_NagC_NanR	b4311
CRP_DnaA_PurR	b2508
CRP_Fis_H-NS	b3723
CRP_GalS	b2151,b0759
PhoB	b2987,b0383,b0241,b1020,b0399,b1597,b3728,b4030,b3453
KdpE	b4513
ArgP	b0031,b2923,b3702,b2234
TorR	b0995,b0996,b3707
ArgP_DnaA_Fis_NrdR	b2234
CRP_Lrp	b0907,b1189,b2659,b4376,b3212
MetJ_MetR	b3828,b3829
RbsR	b3748
ZntR	b3469
CRP_MhpR	b0347
FNR_Fis_IHF_PdhR	b1109
GadE_GadW_GadX	b1493,b3517
H-NS_Lrp	b4314,b2669,b3968
IlvY	b3773,b3774
CpxR_CsgD	b1041
CRP_MelR	b4119,b4118
SoxS	b0850,b0950,b2159,b0684,b1277,b4062,b1852,b1611,b4439,b3908,b1101
CRP_FruR	b2927,b3599,b2415
PhoP	b4242,b4241,b1641,b0154,b1826,b0557,b1130,b1608,b3207
Fis_MarA_MarR_Rob	b1530
MetR_PurR	b2551
CRP_FNR_IHF_NarL	b0904
CRP_IHF_TdcA_TdcR	b3118
ArcA_LexA	b4058,b4059
CadC	b4132

MprA	b2684
FNR_IHF_NarL	b1223,b1224,b0894,b4070,b3365,b0904
IHF_PspF	b1304
BirA	b0774,b0775
FNR_NarL	b0873,b1474,b1674,b2208,b1223,b1224,b0894,b4070,b3365,b4154,b0904
MetR	b4019,b2551,b3828,b3829,b2552
DnaA	b3863,b2508,b3702,b2234,b1415
ArcA_CRP_FadR_OmpR	b2344
UxuR	b4322
EnvY_ModE	b0781
RhaS	b3907,b3904
BolA	b4150,b0839
Nac	b1988,b0336
CRP_IHF_OmpR	b0553
CRP_DgsA_FruR	b2415
TrpR_TyrR	b3161,b0388
CRP_GlpR_IHF	b2240
CRP_TorR	b3707
ArcA_CRP	b0721,b3236,b0720,b2344,b1415,b1101
IHF_Lrp_MarA_Rob_SoxS	b4439
FNR_ModE	b1222,b2208,b0894
CRP_UhpA	b3666
ArcA_LldR	b3603
FadR	b4018,b3495,b2323,b0954,b0221,b2342,b1805,b3846,b2344
OxyR	b2582,b3942,b3961,b2000,b0605,b0849,b0475,b1684,b3518
ArcA_FNR	b0827,b3908,b0733,b2288
Fis_H-NS_Lrp	b3968
IHF_UlaR	b4192,b4193
FhlA_IHF	b2726,b2725
GalR	b2837,b2943,b0759
GcvA_Lrp	b2905
FruR	b1779,b0315,b3916,b1702,b1676,b3403,b4014,b1851,b1241,b3365,b2927,b3599,b2415,b1136,b0733
ArsR	b3501
CRP_FruR_MtlR	b3599
NagC	b3730,b4312,b0678,b0679,b4311,b1817,b1738
IscR	b1706,b2531,b0972,b0156,b1684,b2208
Fis	b4134,b3756,b3278,b2231,b0536,b0405,b3976,b3699,b2397,b3851,b2348,b1032,b4270,b3545,b1739,b2591,b1231,b0673,b0971,b0216,b0201,b2967,b0743,b1274,b3870,b2401,b0244,b3796,b2864,b4370,b4007,b2189,b1530,b3260,b0812,b3968,b1241,b1109,b4070,b3365,b1237,b4000,b3588,b0440,b4111,b3357,b4069,b3723,b3171,b2234,b2288,

	b1101
CRP_CaiF	b0040
Fis_IHF	b3260,b1109,b4070,b3365,b4069,b2288
CspA_Fis_H-NS	b1237
CytR	b3934,b3831,b2143,b0411,b4381,b3363
PspF	b4050,b1303,b1304
PhoB_RstA	b1597
GadE_H-NS_MarA	b3510
Fur	b1252,b2105,b4293,b0593,b0592,b0585,b0591,b4291,b0584,b0590,b2106,b1684,b2552,b3908
CRP_MalI	b1620
ArgP_DnaA	b3702,b2234
CRP_DgsA_NagC	b1817
H-NS_LeuO	b0076,b4365
CRP_H-NS_LacI	b0344
ArgR_NtrC	b1748
FNR_IHF_ModE_NarL	b0894
OmpR	b1634,b0435,b1040,b2215,b0929,b0553,b2344
ArcA_GlcC_IHF	b2979
CRP_IHF_Lrp	b4376,b3212
FhlA	b2713,b4079,b2726,b2725
AraC_CRP	b0396,b1901,b0063,b0064,b2841
NorR	b2709,b2710
CRP_NanR	b3225,b4311
MarA_PurR	b4177
FruR_GntR	b1851
BaeR_CpxR	b2470,b1743,b2074
FNR_OxyR	b3518
CRP_Fis_IHF	b4069
LrhA	b2289,b4313,b1892
ArcA_FNR_FruR_H-NS	b0733
Fur_RcnR	b2106
ArgR_CRP_GadE_IHF_Lrp	b3212
PurR	b1062,b1132,b2553,b0945,b2476,b0523,b2313,b1658,b2499,b2557,b4006,b2551,b4177,b2508,b0032
CRP_CytR_DeoR	b0411,b4381
CRP_DcuR	b3528
Fis_FruR	b1241,b3365
NhaR	b1024,b1482
Lrp	b1243,b4129,b0077,b3460,b0889,b2913,b1482,b3766,b4439,b4314,b2669,b2905,b3968,b2215,b0929,b0907,b1189,b2659,b4376,b3212,b4187

CpxR_OmpR_RstA	b1040
Fis_H-NS	b0812,b3968,b3365,b1237,b3723
ArgR_CRP_Fis	b3171
CsgD	b0385,b1041
FhlA_NarL	b4079
Cbl_CysB_IHF	b0937
MngR	b0731,b0730
AgaR_CRP	b3132
MalI	b1621,b1620
CRP	b4438,b3072,b0331,b3806,b1513,b2096,b3425,b4213,b0330,b3781,b0598,b2509,b3356,b2365,b2150,b1205,b1512,b2801,b3672,b2784,b3666,b3707,b3905,b4030,b3453,b3225,b0678,b0679,b4311,b0347,b4119,b4118,b4034,b4035,b1620,b0907,b1189,b2659,b0125,b3405,b3574,b0124,b3575,b4193,b3118,b1387,b1388,b0553,b4376,b0344,b1892,b3415,b2241,b3426,b2240,b2151,b0759,b2927,b3599,b4000,b3588,b0440,b4111,b3357,b4069,b3723,b2957,b0034,b0904,b1182,b3512,b2508,b3418,b1817,b2415,b3528,b3831,b2143,b0411,b4381,b3363,b1738,b0040,b3172,b3212,b3171,b0721,b3236,b0720,b2344,b1415,b1101,b0396,b1901,b0063,b0064,b2841,b3132
Lrp_NhaR_RcsAB	b1482
CRP_GalR_GalS_H-NS_HU	b0759
IHF_Lrp	b3766,b4439,b2215,b0929,b4376,b3212
GadE_GadW_GadX_H-NS	b3517
ArcA_FNR_Fur_IHF_SoxS	b3908
ArcA	b0313,b0314,b0432,b3603,b4058,b4059,b2979,b1136,b0221,b2342,b1805,b3846,b0827,b3908,b0733,b2288,b0721,b3236,b0720,b2344,b1415,b1101
NrdR	b2673,b4238,b2234
AgaR	b3136,b3131,b3132
NadR	b2574,b0750
MarA	b0578,b4396,b1852,b1611,b4177,b4439,b3510,b1530
RcsAB	b1481,b1482,b1892
CpxR_TyrR	b0754
CusR	b0571,b0572
ArcA_CRP_IHF	b0720
CRP_IHF_YiaJ	b3575
NsrR	b4209
TyrR	b1907,b0048,b0112,b2601,b4054,b3161,b0388,b0754
CRP_GntR	b3415
Fur_IHF_IscR_OxyR	b1684
FruR_IHF_IclR	b4014

FNR_NikR-Ni	b3476
CRP_EvgA_GadE_YdeO	b3512
Cbl_CysB	b0365,b0937
H-NS_LrhA	b4313,b1892
FhlA_IHF_ModE	b2725
NtrC	b0811,b1748
IHF_NorR	b2710
ArgR_IHF_PurR_RutR	b0032
QseB	b3025
FlhDC	b1922,b1880,b1073,b0655,b1904,b1944
MalT	b3417,b4034,b4035
ZraR	b4003,b4002
CRP_RhaR	b3905
TreR	b4240
FabR_FadR	b2323,b0954
ArcA_CRP_DgsA_Fis_SoxS	b1101
ArcA_FadR	b0221,b2342,b1805,b3846,b2344
FNR_Fur_MetR	b2552
CRP_ChbR_NagC	b1738
DgsA	b1594,b3418,b1817,b2415,b1101
CaiF	b0041,b0040
ArcA_CRP_DnaA	b1415
CRP_IHF_UlaR	b4193
ModE	b0763,b2725,b1222,b2208,b0894,b0781,b4381
CRP_IHF	b0125,b3405,b3574,b0124,b3575,b4193,b3118,b1387,b1388,b0553,b4376,b2240,b4069,b0904,b3212,b0720
GadE_H-NS	b3511,b3510,b3517
FNR_IscR_ModE_NarL_NarP	b2208
AcrR_EnvR_Rob	b0463
Ada_Lrp	b4187
SoxR	b4063,b4062,b1611
AcrR	b0464,b0463
CpxR_IHF_Lrp_OmpR	b2215,b0929
IHF_PhoB	b3728
FNR	b1210,b2579,b1334,b0113,b3518,b3476,b0873,b1474,b1674,b1222,b2208,b1223,b1224,b0894,b2552,b1109,b4070,b3365,b4154,b2957,b0034,b0904,b1182,b0827,b3908,b0733,b2288
CRP_DgsA	b3418,b1817,b2415,b1101
ArcA_FruR	b1136,b0733
AllS	b0517

ArgR_CRP	b3172,b3212,b3171
EvgA	b2369,b2368,b1501,b2374,b2375,b1500,b3512
CueR	b0484,b0123
XylR	b3566,b3565
DcuR	b4123,b4154,b3528
H-NS_IHF_NagC_NanR	b4312
CRP_FNR_H-NS_SlyA	b1182
FNR_Fis_IHF_NarL_NarP	b4070,b3365
FNR_NarL_NarP	b1474,b1674,b2208,b4070,b3365
CRP_CytR	b3831,b2143,b0411,b4381,b3363
CRP_PhoB	b4030,b3453
NemR	b1649
FNR_Fis_FruR_H-NS_IHF_NarL_NarP	b3365
CynR	b0338,b0339
CRP_MalT	b4034,b4035
NarL	b4139,b4125,b4079,b0873,b1474,b1674,b2208,b1223,b1224,b0894,b4070,b3365,b4154,b0904
RutR	b1013,b1012,b0032
GadE	b1951,b3511,b3510,b1493,b3517,b3512,b3212
MarA_Rob_SoxS	b1852,b1611,b4439
MetJ	b3008,b4013,b3828,b3829
AllR	b0505,b0507,b0504
IHF	b3461,b2764,b3544,b4026,b4117,b4192,b1304,b3728,b2710,b3766,b4439,b4312,b1684,b4014,b3260,b2726,b2725,b1223,b1224,b0894,b1109,b4070,b3365,b2215,b0929,b0937,b0125,b3405,b3574,b0124,b3575,b4193,b3118,b1387,b1388,b0553,b4376,b2240,b4069,b0904,b2221,b0032,b3212,b2979,b3908,b2288,b0720
GlpR	b3927,b2241,b3426,b2240
ArcA_FNR_Fis_IHF	b2288
Zur	b1857,b1858
MntR	b2392
SoxR_SoxS	b4062,b1611
Rob	b2237,b1852,b1611,b4439,b1530,b0463
CRP_IHF_PaaX	b1387,b1388

H-NS	b3323,b4364,b2677,b3324,b3338,b1967,b1259,b0564,b4314,b2669,b4313,b0076,b4365,b4312,b3511,b3510,b3517,b0812,b3968,b3365,b1237,b0344,b1892,b0759,b3723,b1182,b0733
CRP_CytR_DeoR_ModE	b4381
CpxR	b1535,b1890,b1016,b0161,b3095,b3859,b1902,b0461,b0970,b1113,b4484,b1846,b4355,b0441,b2580,b0754,b1040,b2215,b0929,b1041,b3363,b2470,b1743,b2074
ArgR	b0864,b3237,b3957,b3359,b2818,b3958,b0273,b0860,b4254,b1748,b0032,b3172,b3212,b3171
CRP_H-NS_HdfR_LrhA_RcsAB	b1892
CRP_NagC	b0678,b0679,b4311,b1817,b1738
HipB	b1508

References

1. Lemmens K, De Bie T, Dhollander T, De Keersmaecker SC, Thijs IM, Schoofs G, De Weerdt A, De Moor B, Vanderleyden J, Collado-Vides J, Engelen K, Marchal K: **DISTILLER: a data integration framework to reveal condition dependency of complex regulons in *Escherichia coli*.** *Genome Biol* 2009, **10**:R27.1-R27-13.

2. Gama-Castro S, Jimenez-Jacinto V, Peralta-Gil M, Santos-Zavaleta A, Penaloza-Spinola MI, Contreras-Moreira B, Segura-Salazar J, Muniz-Rascado L, Martinez-Flores I, Salgado H, Bonavides-Martinez C, Abreu-Goodger C, Rodriguez-Penagos C, Miranda-Rios J, Morett E, Merino E, Huerta AM, Trevino-Quintanilla L, Collado-Vides J: **RegulonDB: gene regulation model of Escherichia coli K-12 beyond transcription,**

active (experimental) annotated promoters and Textpresso navigation. *Nucleic Acids Res* 2008, **36**: D120-124.
