# Supplementary material for: Query-based biclustering of gene expression data using Probabilistic Relational Models
Source: BMC Bioinformatics. 2011 Feb 15;12(Suppl 1):S37. doi: 10.1186/1471-2105-12-S1-S37 (PMC3044293; doi:10.1186/1471-2105-12-S1-S37)
Supplement: Additional File 4 — Influence of parameter settings It contains an analysis of the influence of two parameters that are influencial in a query-based setting on the obtained bicluster result for five representative seed sets. [file 1471-2105-12-S1-S37-S4.pdf]

Additional File 4 - Influence of parameter settings

In this section, we chose five representative seed sets to analyze the influence of two parameters that are influencial in a query-based setting on the obtained bicluster result.

4.1 Parameter $\log\frac{\pi_{bicl}}{\pi_{bgr}}$:

The user-defined parameter $\log\frac{\pi_{bicl}}{\pi_{bgr}}$ indicates how many times more likely it must be that an expression value is part of the bicluster distribution compared to being part of the background distribution before it is actually assigned to that bicluster. To determine the effect of this parameter on the bicluster results, we tested a sweep over the parameter $\log\frac{\pi_{bicl}}{\pi_{bgr}}$, ranging from -3.0 until 0 with steps of 0.1. As shown in Figure 4.1, for all the five examples, decreasing the $\log\frac{\pi_{bicl}}{\pi_{bgr}}$ value results in biclusters with a gradually higher STD-across but at the expense of a higher STD-within. Biclusters will thus differ more from the background (higher STD-across) but the genes in the bicluster will be less tightly coexpressed (higher STD-within). Good biclusters should keep a balance between the STD-across and STD-within.

The number of genes and conditions in the biclusters is proportional to the $\log\frac{\pi_{bicl}}{\pi_{bgr}}$- value: larger parameter values correspond to larger biclusters, as shown in Figures 4.2 and 4.3.

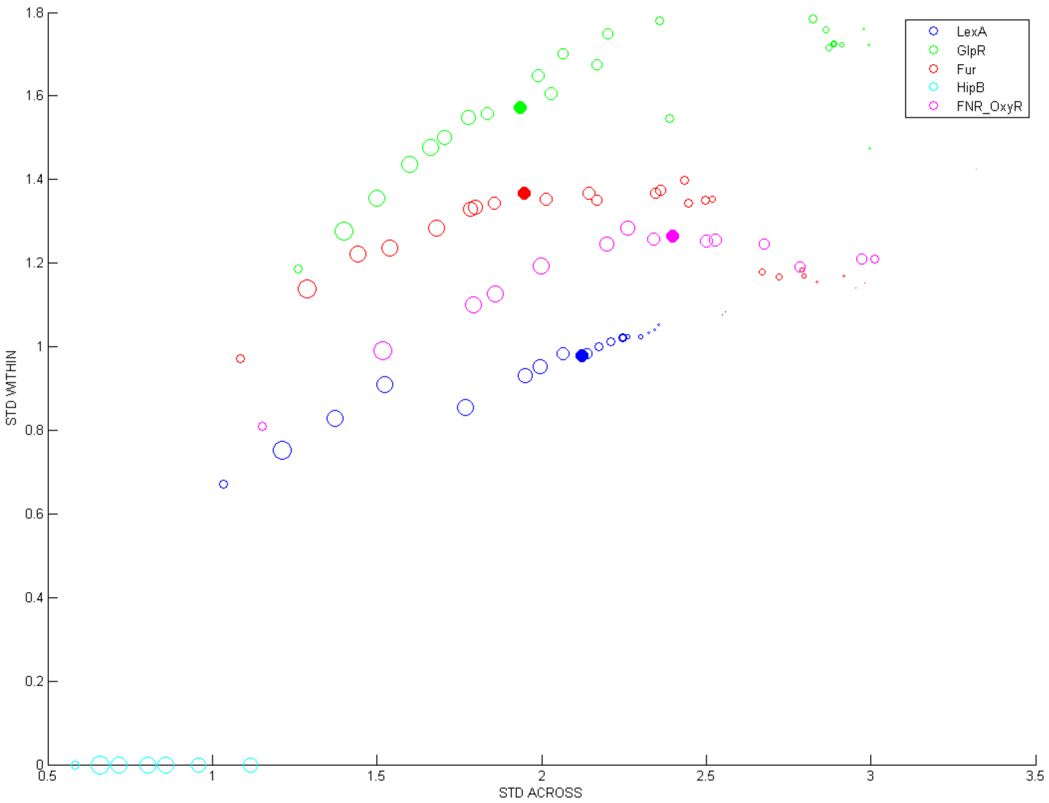

Figure 4.1: Variation of the bicluster quality as a function of different $\log \frac{\pi_{bicl}}{\pi_{bgr}}$ values.

Different colors correspond to different seed sets. The size of the circles corresponds to the parameter value. The filled circles correspond to the value of the parameter used in all experiments. High quality biclusters have a low STD-within and a high STD-across.

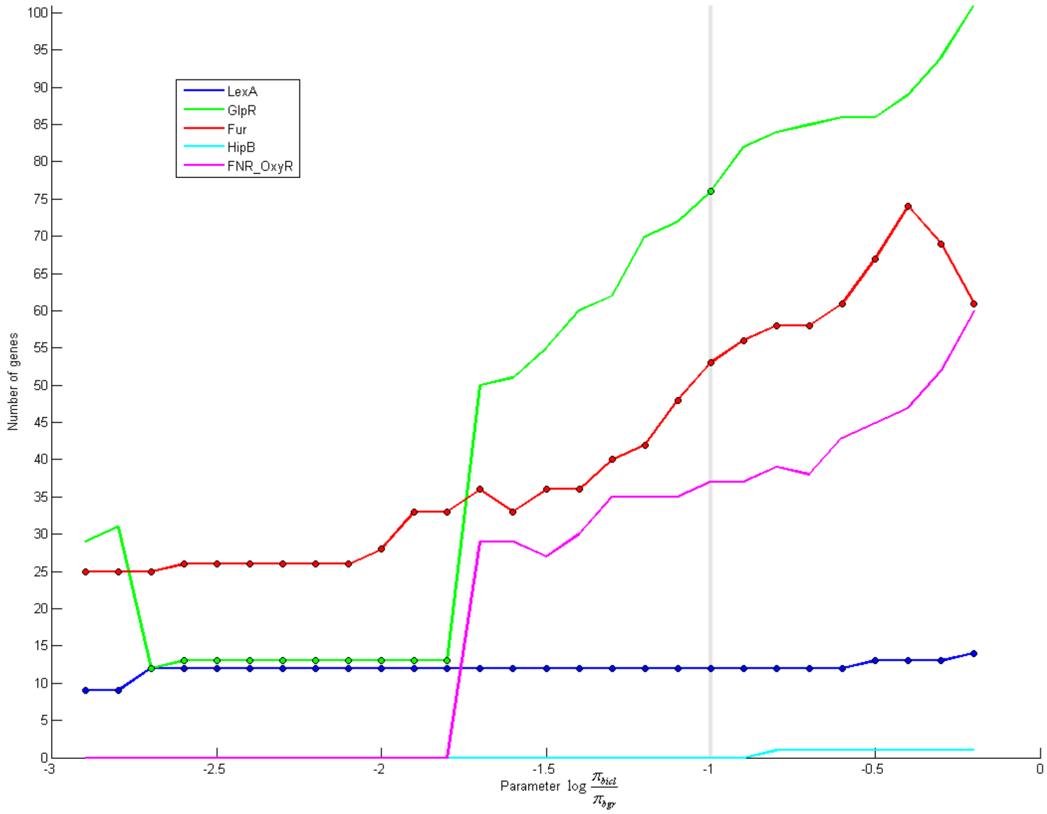

Figure 4.2: Variation of the number of genes in the bicluster as a function of different $\log \frac{\pi_{bicl}}{\pi_{bgr}}$ values.

Different colors correspond to different seed sets. The filled points on the curves correspond to the biclusters with the same enriched functions as the seed genes. The grey vertical line corresponds to the value of the parameter used in all experiments.

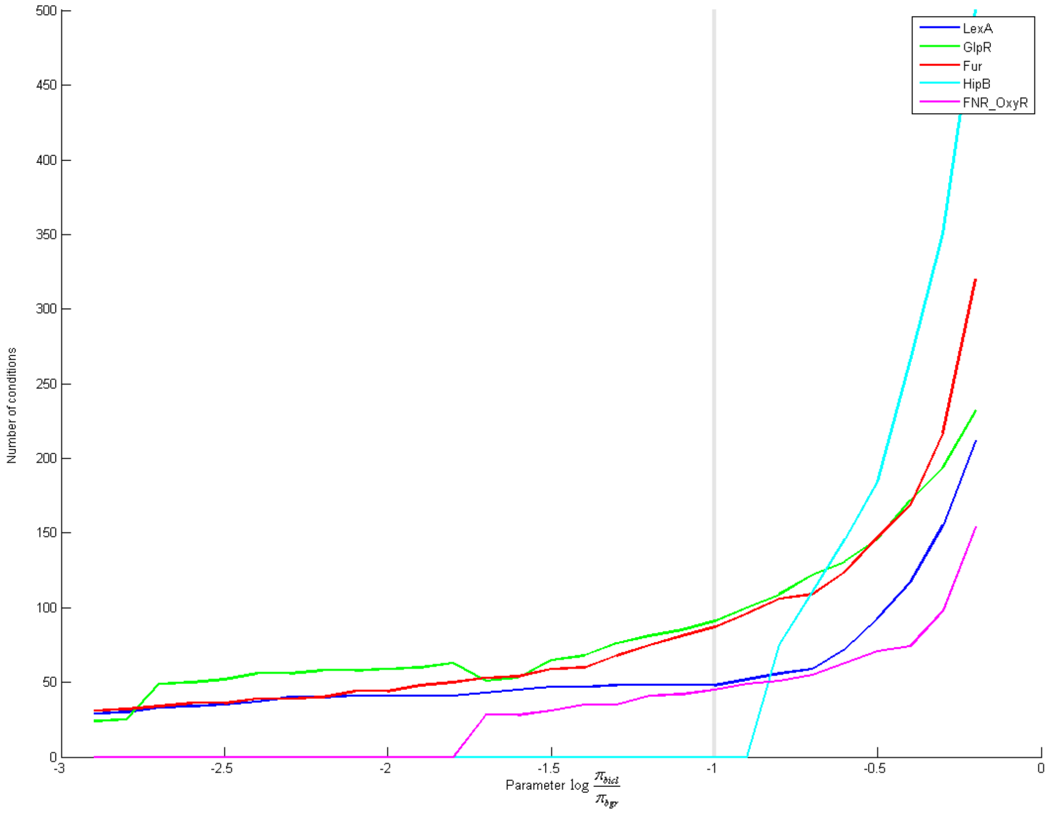

Figure 4.3: Variation of the number of conditions in the bicluster as a function of different $\log \frac{\pi_{bicl}}{\pi_{bgr}}$ values.

Different colors correspond to different seed sets. The grey vertical line corresponds to the value of the parameter used in all experiments.

4.2 Parameter f_{bcl}:

The prior standard deviation $\sigma_{a,b}^{0}$ is chosen to be a fraction f_{bcl} of the background standard deviation and thus f_{bcl} determines the tightness of the bicluster profile.

To test its influence, the parameter f_{bcl} was varied in a range from 0.2 to 3 with steps of 0.1. Figure 4.4 illustrates how the bicluster quality changes as a function of f_{bcl}.. Decreasing f_{bcl} results in biclusters of increasing quality as measured with a decreasing STD-within and increasing STD-across (biclusters with tightly coexpressed genes of which the profiles are well distinguished from the background). This high quality of the biclusters expression profiles comes at the expense of the cluster size as is shown in Figure 4.5: too small f_{bcl} values result in 'empty' biclusters or biclusters with too few genes.

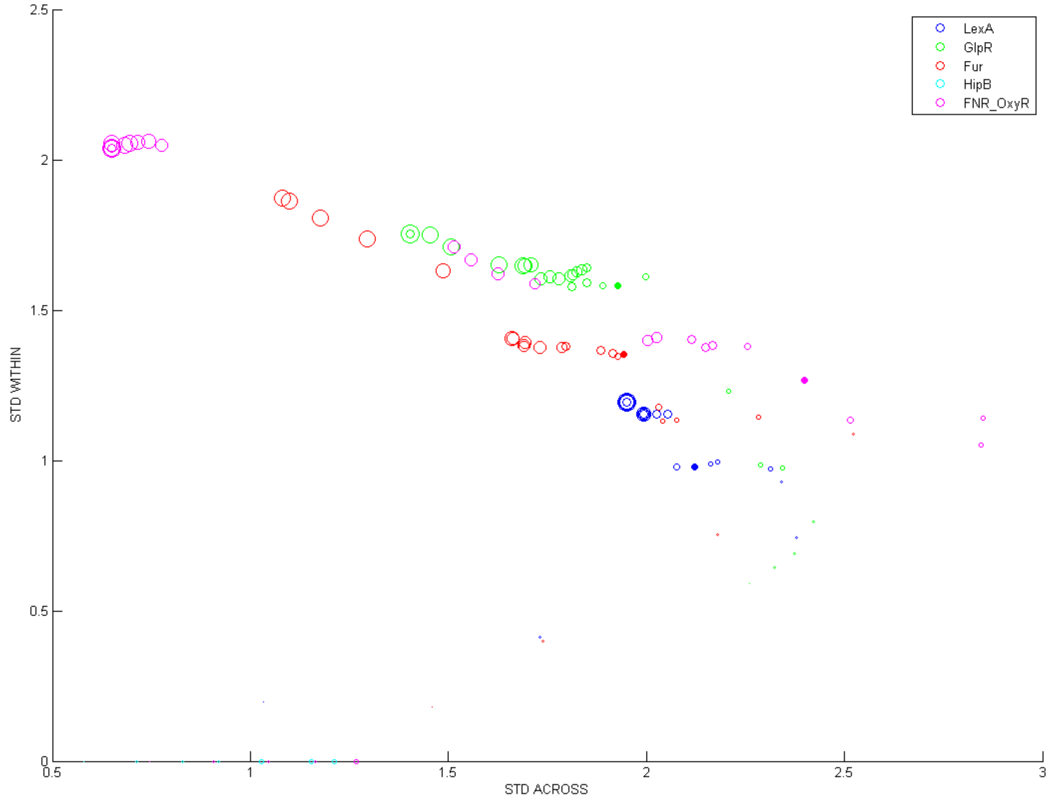

Figure 4.4: **Variation of the bicluster quality as a function of different f_{bcl} values**.

Different colors correspond to different seed sets. The size of the circles corresponds to the value of parameter. The filled circles correspond to default values of the parameters used in all experiments.

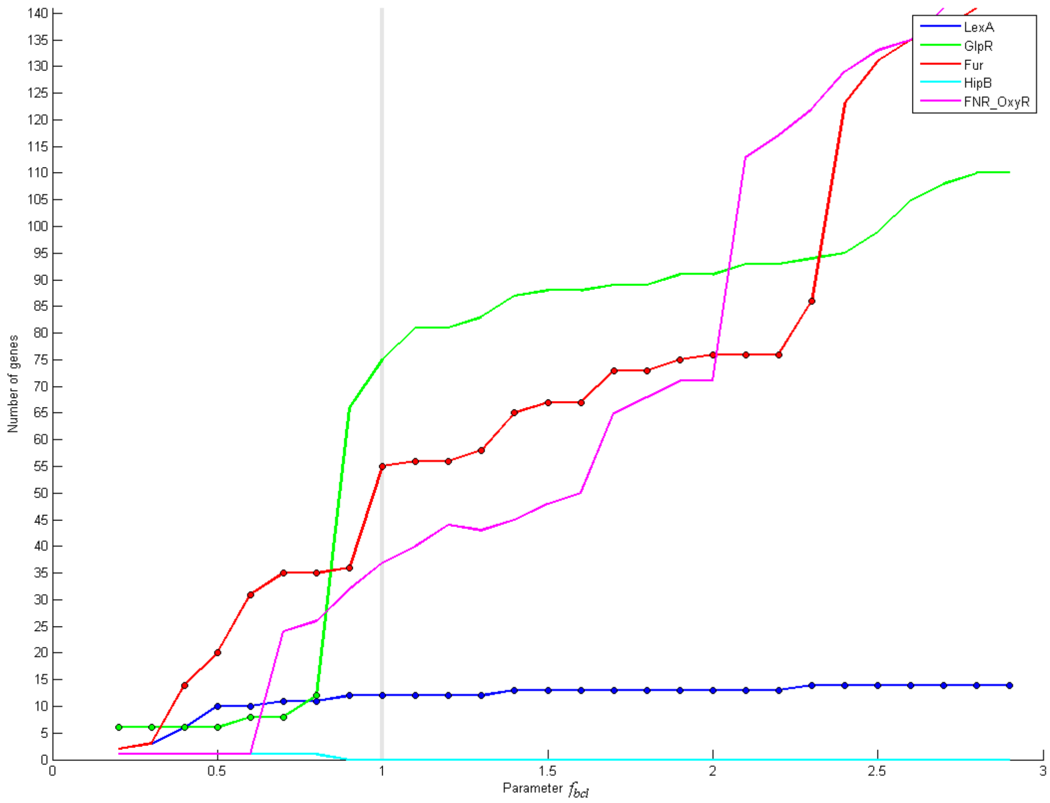

Figure 4.5: Variation of the number of genes in the bicluster as a function different f_{bcl} values.

Different colors correspond to different seed sets. The filled points on the curves correspond to the biclusters with the same enriched functions as the seed genes. The grey vertical line corresponds to the value of the parameter used in all experiments.

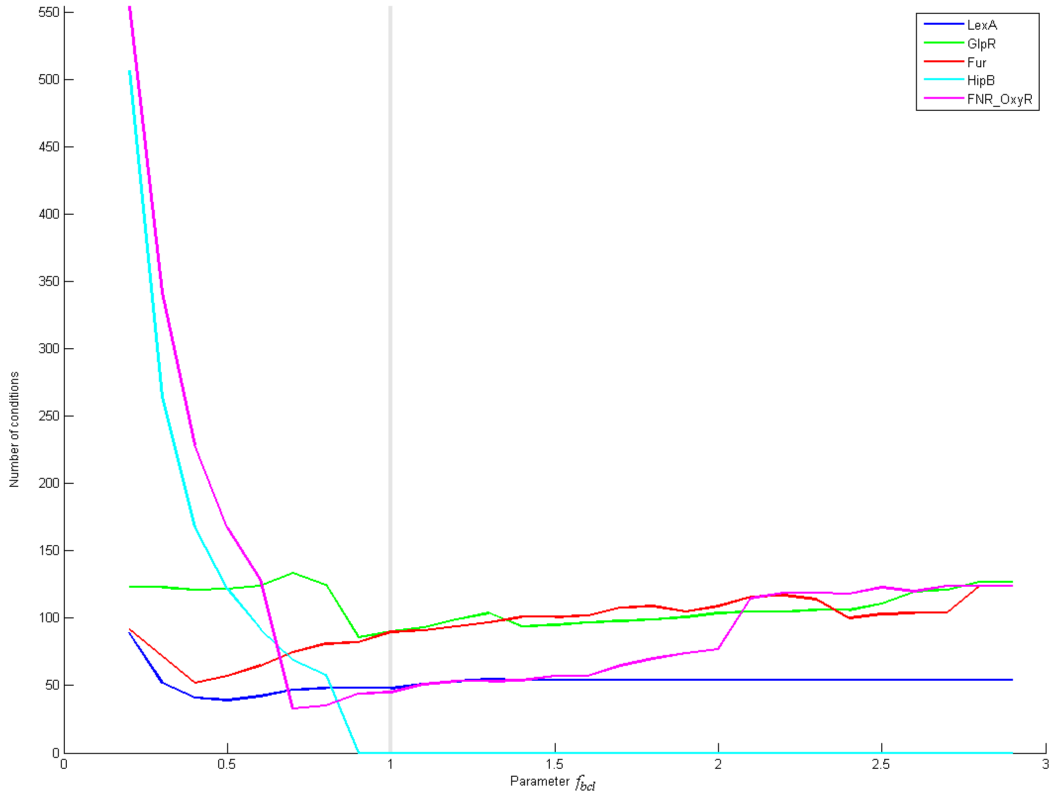

Figure 4.6: Number of conditions in the bicluster as a function of different f_{bcl} values. Different colors correspond to different seed sets. The grey vertical line corresponds to the value of the parameter used in all experiments.
