# Supplementary material for: Query-based biclustering of gene expression data using Probabilistic Relational Models
Source: BMC Bioinformatics. 2011 Feb 15;12(Suppl 1):S37. doi: 10.1186/1471-2105-12-S1-S37 (PMC3044293; doi:10.1186/1471-2105-12-S1-S37)
Supplement: Additional File 5 — Running parameters of query-based biclustering tools It contains the parameter settings for all query-based biclustering algorithms (ProBic, QDB and ISA) that were used to run the experiments performed in the article. [file 1471-2105-12-S1-S37-S5.pdf]

Additional File 5 - Running parameters of query-based biclustering tools

5.1 *Pro*Bic

For the experiments performed, a number of parameters were set in advance. These include the $P_1(g.B)$ parameter, which was set to -0.1 for all genes. The $P_2(g.B_b)$ parameter was set to 0.5 for all genes, meaning that they have an equal probability of belonging to a bicluster *b* or the background. Similarly, the value of $P(a.B_b)$ was set to 0.5 for all arrays. The $\log \frac{\pi_{bicl}}{\pi_{bgr}}$ parameter to decrease model complexity was set to -1. The choice of hyperparameters that determine the Normal-Inverse-χ^2distributions, chosen as prior on the model parameters, was as follows: the prior background distribution was parameterized by (0, 0.000001, 1, 1) for $(\mu_0,\kappa_0,\nu_0,\sigma^2_0)$ respectively. These values result in an extremely uninformative (*i.e.*, non-influential) prior as the background distribution should follow from the data itself. The prior bicluster distribution was parameterized as follows for the four parameters respectively: (μ_a^{query}, 100 000, 1000, $f_{bcl}*\sigma_a^{bgr}$). By choosing the prior mean μ_0 as the sample average μ_a^{query} for the expression values defined by the set of seed genes for each array *a*, the algorithm will identify a bicluster around the expression profile of these seed genes. The prior standard deviation $\sigma_{a,b}^0$ was chosen equal to the background standard deviation σ_a^{bgr} (f_{bcl} = 1).

Parameter κ_0 was chosen high relatively to the other hyperparameters in order to force the variance on the prior mean to be small and preventing the bicluster from drifting too far away from the seed profile. The parameter ν_0, which determines the relative weight of the prior to the data, was set to 1000 as this seemed to keep a good balance between the prior and the data.

5.2 QDB

All simulations shown for Query-Driven Biclustering (QDB) were obtained using the resolution sweep approach. We linearly increased the variance prior parameter for each condition from 0 to the corresponding background variance over 100 iterations. Other parameters were left to their default values suggested by the author. To automatically detect the resolutions of interest, we identified the local maxima in the Akaike Information Criterion (AIC) described in the paper of D'Hollander *et al*. (2007) [1].

5.3 ISA

ISA (Iterative Signature Algorithm) can be used in a query-based setting by applying non-random seeds. In this case the algorithm is deterministic and can be considered as a semi-supervised approach. The two main parameters of ISA are the condition and gene thresholds. Bergmann *et al*. (2003) [2], suggested values for the gene threshold varying from 2 to 4. We applied a parameter sweep between these thresholds and selected 3 as the optimal value (where biclusters were obtained that showed the best expression quality and the highest number of functionally enriched biclusters). The condition threshold was taken 2, as suggested by the authors (Bergmann *et al*., 2003) [2].

References

1. Dhollander T, Sheng Q, Lemmens K, De Moor B, Marchal K, Moreau Y: **Query-driven module discovery in microarray data**. *Bioinformatics* 2007, **23**:2573-2580.
2. Bergmann S, Ihmels J, Barkai N: **Iterative signature algorithm for the analysis of large-scale gene expression data**. *Physical review 2003*, **E.67**:031902-1-031902-18.
