# Supplementary material for: Query-based biclustering of gene expression data using Probabilistic Relational Models
Source: BMC Bioinformatics. 2011 Feb 15;12(Suppl 1):S37. doi: 10.1186/1471-2105-12-S1-S37 (PMC3044293; doi:10.1186/1471-2105-12-S1-S37)
Supplement: Additional File 6 — Behavior of the different algorithms towards seed genes It contains an additional table to the section ‘Behavior towards seed genes’, that displays the number of bicluster results for respectively ProBic, QDB, ISA belonging to different categories related to the seed genes. [file 1471-2105-12-S1-S37-S6.pdf]

Additional File 6 - Behavior of the different algorithms towards seed genes

The table displays the number of bicluster results for respectively *Pro*Bic, QDB, ISA that contain ‘Empty’ biclusters, ‘Drift away’ biclusters (clusters containing one or more genes, but none of the seed genes), ‘Only full seed set’ biclusters (containing all initial seed genes but without additional genes), ‘Part of the seed set’ biclusters (containing only a part of the initial seed genes but without additional genes), ‘Part of the seed set and additional genes’ biclusters (containing a part of the initial set of seed genes together with additional genes) and ‘Full seed set and additional genes’ biclusters (containing all seed genes together with additional genes).

	***Pro*Bic**	**QDB**	**ISA**
**Empty**	4	49	0
**Drift away**	15	15	76
**Only full seed set**	51	51	0
**Only part of seed set**	10	0	0
**Part of seed set and additional genes**	80	17	96
**Full seed set and additional genes**	65	93	53
