# Supplementary material for: Query-based biclustering of gene expression data using Probabilistic Relational Models
Source: BMC Bioinformatics. 2011 Feb 15;12(Suppl 1):S37. doi: 10.1186/1471-2105-12-S1-S37 (PMC3044293; doi:10.1186/1471-2105-12-S1-S37)
Supplement: Additional File 7 — Robustness of ProBic, QDB and ISA to noisy seed genes of intermediate quality It contains additional figures to the section ‘Difference in handling noisy seed genes’, that depict the performance of respectively ProBic, QDB and ISA in case of noisy genes added to three seed sets of intermediate quality. [file 1471-2105-12-S1-S37-S7.pdf]

**Additional File 7 - Robustness of *Pro*Bic, QDB and ISA to noisy seed genes of intermediate quality**

Analysis of biclusters, obtained by the different algorithms after biclustering in the presence of an increasing number of random genes (1 = 20%, 2 = 40%, 3 = 60% and 4 = 80% of random genes), added to the true seed set of three seed sets of intermediate quality (panel a: FadR, panel b: NarL, panel c: OmpR). The procedure of adding random sets was repeated 100 times for each seed set. Legend is as in Figure 3 of the main text.

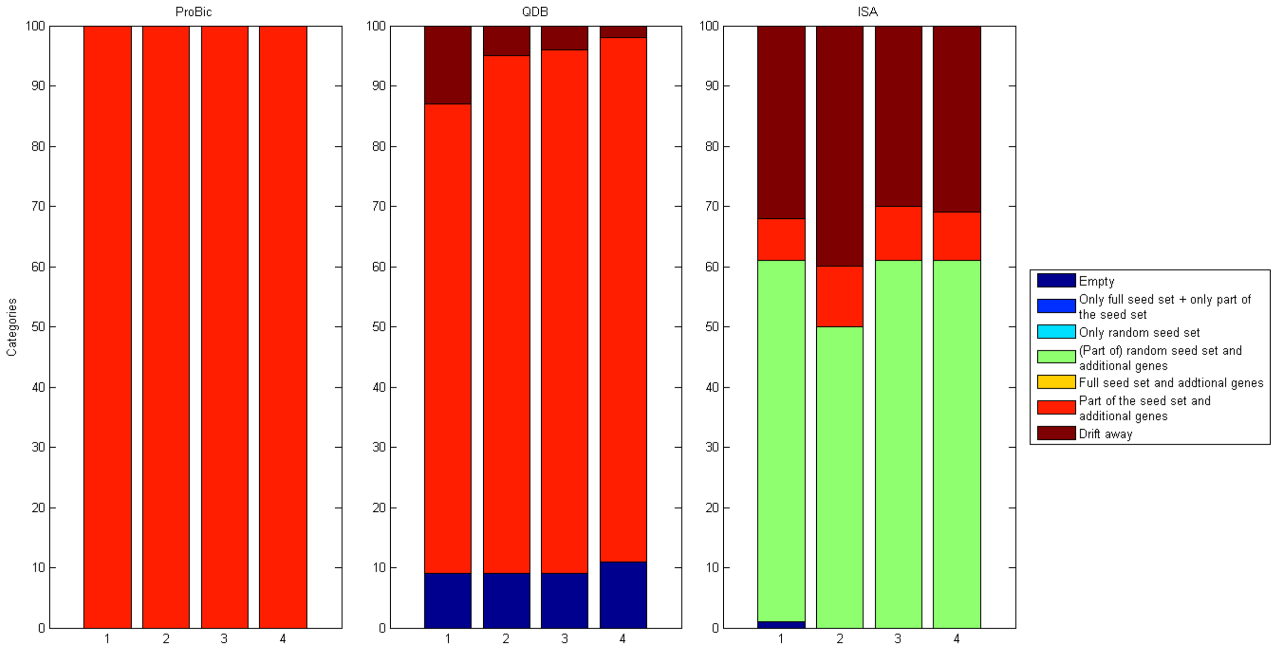


(a)

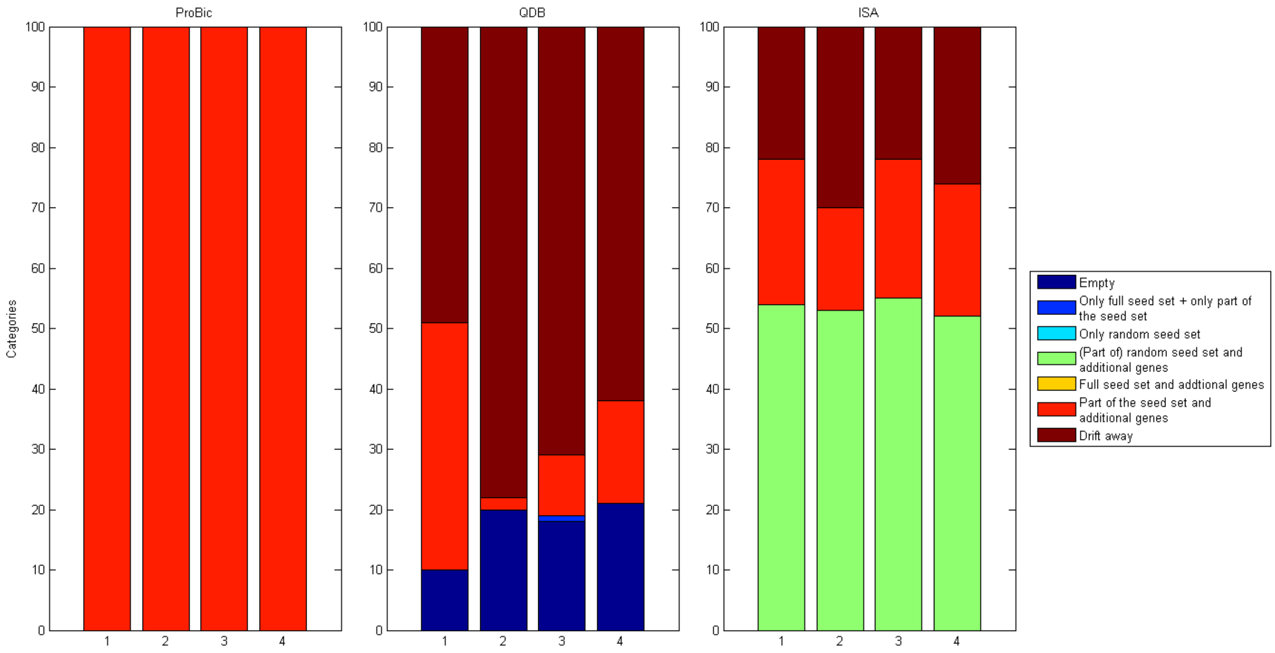
ProBic
QDB
ISA
Categories
Empty
Only full seed set + only part of the seed set
Only random seed set
(Part of) random seed set and additional genes
Full seed set and addtional genes
Part of the seed set and additional genes
Drift away

(b)

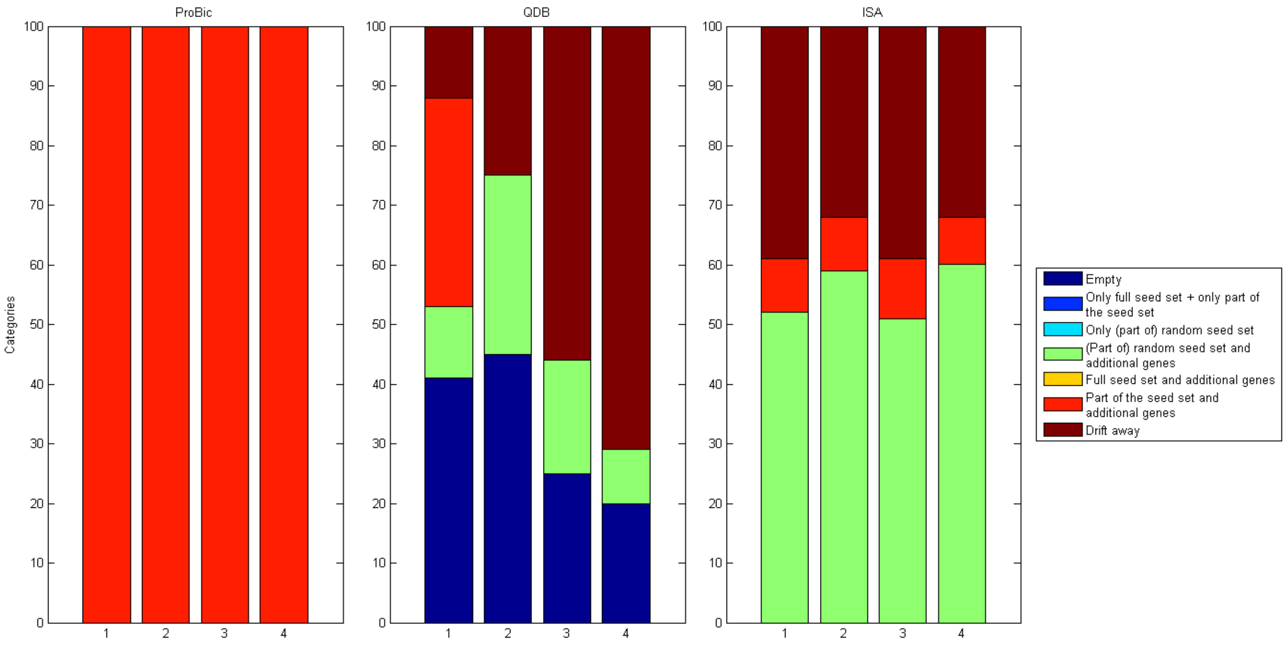
ProBic
QDB
ISA
Categories
Empty
Only full seed set + only part of the seed set
Only (part of) random seed set
(Part of) random seed set and additional genes
Full seed set and additional genes
Part of the seed set and additional genes
Drift away

(c)
