# Supplementary material for: Query-based biclustering of gene expression data using Probabilistic Relational Models
Source: BMC Bioinformatics. 2011 Feb 15;12(Suppl 1):S37. doi: 10.1186/1471-2105-12-S1-S37 (PMC3044293; doi:10.1186/1471-2105-12-S1-S37)
Supplement: Additional File 8 — Recall and ‘enrichment’ of the biclusters in a cross-validation experiment It contains an additional figure to the section ‘Cross-validation for identification of known targets of TF(s)’, that depicts the recall and enrichment scores for the different seed sets, for each of the three query-based biclustering approaches. [file 1471-2105-12-S1-S37-S8.pdf]

Additional File 8 – Recall and 'enrichment' of the biclusters in a cross-validation experiment

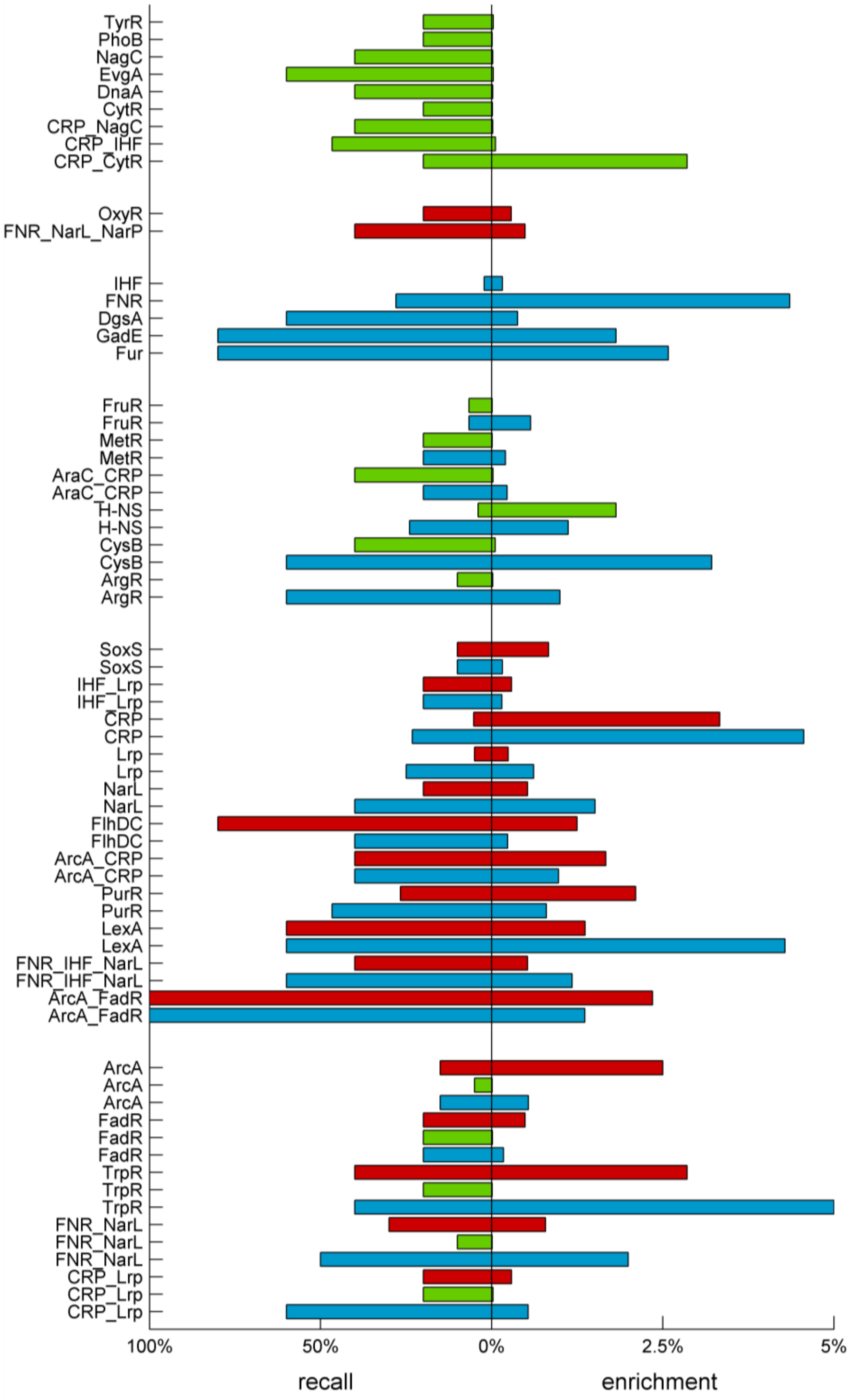

Results of the cross-validation experiment performed for respectively *Pro*Bic (blue), QDB (green) and ISA (red). (x-axis left) Recall: the percentage of genes of the validation set that was retrieved in the biclusters; (x-axis right) 'Enrichment': the percentage of validation genes found in the biclusters to the total number of genes in the bicluster. Scores were averaged over five cross-validations. (y-axis) The regulons' associated TF(s) for which this experiment was performed.
